# M6A Modified miR‐31‐5p Suppresses M1 Macrophage Polarization and Autoimmune Dry Eye by Targeting P2RX7

**DOI:** 10.1002/advs.202415341

**Published:** 2025-03-11

**Authors:** Lu Zhao, Xuejia Li, Min Gao, Lin Liu, Binyun Ma, Xun Liu, Jiachen Zhang, Ruoxuan Liu, Bei Du, Ruihua Wei, Hong Nian

**Affiliations:** ^1^ Tianjin Key Laboratory of Retinal Functions and Diseases Tianjin Branch of National Clinical Research Center for Ocular Disease Eye Institute and School of Optometry Tianjin Medical University Eye Hospital Tianjin 300384 China; ^2^ Department of Medicine/Hematology Keck School of Medicine of the University of Southern California Los Angeles CA 90033 USA

**Keywords:** m6A, macrophage polarization, miR‐31‐5p, p2x7 receptor, sjogren's syndrome dry eye

## Abstract

The dysregulation of the M1/M2 macrophage balance plays a pivotal role in autoimmune diseases. However, the interplay between microRNAs (miRNAs) and N6‐methyladenosine (m6A) modulation in regulating this balance remains poorly understood. Here, a significant reduction in miR‐31‐5p levels is observed in the lacrimal glands of rabbit autoimmune dacryoadenitis and the peripheral blood mononuclear cells (PBMCs) of Sjögren's syndrome (SS) dry eye patients. Overexpression of miR‐31‐5p exhibits preventive and therapeutic effects on rabbit autoimmune dacryoadenitis. Further investigation revealed that miR‐31‐5p overexpression significantly restored the M1/M2 macrophage balance both in vivo and in vitro. Mechanistically, miR‐31‐5p directly targets the P2x7 receptor (P2RX7), leading to the inactivation of p38 mitogen‐activated protein kinases (MAPK) signaling and reduced expression of M1 markers. Furthermore, methylated RNA immunoprecipitation and luciferase reporter assays demonstrated that fat mass and obesity‐associated protein (FTO)‐mediated m6A demethylation, which sustains pri‐miR‐31 stability, is responsible for the decreased miR‐31‐5p levels in autoimmune dry eye. Notably, PBMC samples from SS dry eye patients further support the link between reduced miR‐31‐5p levels and M1 macrophage activation observed in rabbits. Overall, these data highlight the critical role of the FTO/miR‐31‐5p/P2RX7/p38 MAPK axis in autoimmune inflammation, suggesting their potential as therapeutic targets for autoimmune dry eye.

## Graphical abstract

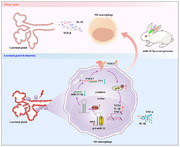

## 1 Introduction

Sjogren's syndrome (SS) dry eye is an intractable autoimmune disease characterized by lymphocyte infiltration and dysfunction in the lacrimal glands (LGs), which may ultimately lead to visual impairment.^[^ ^1^, ^2^ ^]^ Although autoreactive T cells play an important role in SS dry eye pathogenesis,^[^ ^2^, ^3^ ^]^ the implication of macrophages in the development of SS dry eye has been gaining ground recently.^[^ ^4^ ^]^ Indeed, the number of macrophages aggregating in LGs was found to be positively correlated with the infiltration grade and biopsy focus scores in SS patients.^[^ ^5^, ^6^ ^]^

Macrophages exhibit different phenotypes that exert distinct functions on the development of autoimmune diseases. M1 macrophages, marked by nitric oxide synthase 2 (NOS2), exert pro‐inflammatory effects through promoting the production of inflammatory factors, including interleukin (IL)‐1β and tumour necrosis factor (TNF)‐α.^[^ ^7^, ^8^ ^]^ On the contrary, M2 macrophages, which highly express arginase 1 (Arg1) and mannose receptor (CD206), can facilitate the resolution of inflammation and tissue repair via secreting anti‐inflammatory mediators, such as IL‐10 and transforming growth factor (TGF)‐β.^[^ ^7^, ^9^ ^]^ Recent studies have demonstrated that M1/M2 imbalance plays a pathogenic role in SS pathogenesis.^[^ ^10^, ^11^ ^]^ Significant increases in M1 macrophages and associated pro‐inflammatory cytokines have been found in the peripheral blood of SS patients, potentially fueling the chronic inflammatory process within the exocrine glands.^[^ ^11^ ^]^ Our published works have verified that skewing macrophages to an M2 phenotype can contribute to alleviating autoimmune dacryoadenitis.^[^ ^4^, ^12^ ^]^ However, the molecular mechanisms regulating the M1/M2 balance in autoimmunity remain largely unknown.

Named after their small size of ≈21–25 nucleotides, microRNA (miRNAs) are non‐coding RNAs that negatively regulate gene expression at the post‐transcriptional level.^[^ ^13^ ^]^ A miRNA can target a large network of mRNAs to affect diverse aspects of physical and pathological processes, including macrophage polarization.^[^ ^14^ ^]^ By conducting miRNAs sequencing analysis of LGs between a rabbit dry eye model and normal controls, we found that miR‐31‐5p was significantly downregulated in the LGs of dry eye rabbits. Emerging evidence has highlighted the controversial role of miR‐31 in autoimmune disorders. miR‐31 promoted epidermal hyperplasia and exacerbated psoriatic inflammation in psoriasis,^[^ ^15^ ^]^ worsened experimental autoimmune encephalomyelitis by repressing G protein‐coupled receptor 5 (GPRC5).^[^ ^16^ ^]^ However, overexpression of miR‐31 has been found to attenuate murine allergic rhinitis via suppressing IL‐13‐induced nasal epithelial inflammatory responses,^[^ ^17^ ^]^ and to relieve neuropathic pain by inhibiting TNF receptor associated factor 6 (TRAF6)‐mediated neuroinflammation.^[^ ^18^ ^]^ However, to our knowledge, no prior study has addressed whether and how miR‐31 regulates the pathogenesis of autoimmune dry eye.

N6‐methyladenosine (m6A) methylation, a ubiquitous RNA modification of eukaryotic RNA, participates in RNA transcription, translation, and stability.^[^ ^19^, ^20^ ^]^ m6A is dynamically regulated by methyltransferases (methyltransferase like (METTL)3 and METTL14), demethylases (fat mass and obesity‐associated protein (FTO) and AlkB homolog 5 (ALKBH5)), and methylated reading proteins (YTH domain‐containing (YTHDC)1/2 and YTH domain‐containing family protein (YTHDF)1/2/3).^[^ ^20^, ^21^ ^]^ Aberrant expression of these m6A regulators has been implicated in the pathogenesis of autoimmune disorders, including SS.^[^ ^22^, ^23^, ^24^ ^]^ In recent years, m6A modification in miRNAs targeting oncogenes or tumor suppressors has been reported to play a pivotal role in cancer progression.^[^ ^25^ ^]^ Through repressing immunoregulators, such as dual‐specificity phosphatase 16 (DUSP16) and protein kinase AMP‐Activated catalytic subunit alpha 2 (PRKAA2), m6A‐regulated miRNAs have been found to aggravate experimental autoimmune uveitis (EAU) and osteoarthritis.^[^ ^26^, ^27^ ^]^ Nevertheless, the role of m6A modification in SS dry eye remains unclear. Moreover, the function of m6A‐regulated miRNAs in autoimmune dry eye needs further exploration.

In this study, we demonstrated that FTO‐regulated miR‐31‐5p, significantly downregulated in peripheral blood mononuclear cells (PBMCs) of SS dry eye patients and in LGs of rabbit autoimmune dacryoadenitis, an animal model of autoimmune dry eye which can closely mimic human SS dry eye disease,^[^ ^28^, ^29^ ^]^ was critical for autoimmune dry eye progression. By targeting the p2x7 receptor (P2RX7), miR‐31‐5p inactivated p38 mitogen‐activated protein kinases (MAPK) signaling, leading to suppressed M1 activation and alleviated autoimmune dacryoadenitis.

## 2 Results

### 2.1 miR‐31‐5p is Significantly Decreased Both in LGs of Rabbit Autoimmune Dacryoadenitis and in PBMCs of Human SS Dry Eye Patients

**Figure 1:**
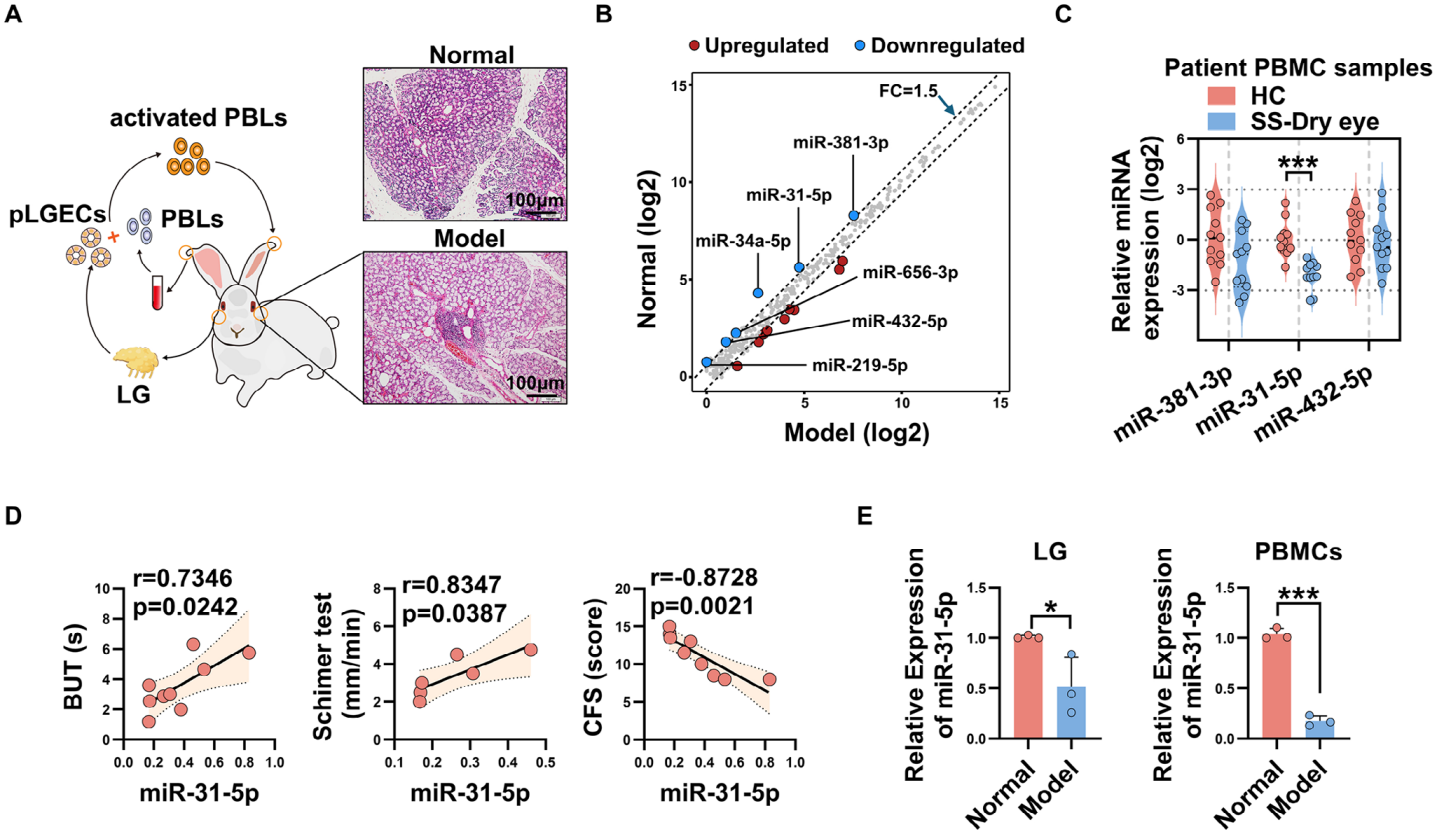
miR‐31‐5p is decreased in LGs of rabbit autoimmune dacryoadenitis and PBMCs of human SS dry eye patients. A) Small RNA sequencing workflow schematic. Autoimmune dacryoadenitis in rabbits was induced by transferring activated PBLs. At 6 weeks post‐induction, LGs of normal and model group rabbits were collected for small RNA sequencing (*n* = 3/group). B) Small RNA‐sequencing‐based scatter plot showing the differentially expressed miRNAs in LGs isolated from rabbit autoimmune dacryoadenitis relative to those from normal controls. Red dots indicate upregulated miRNAs and blue dots represent downregulated miRNAs C) Dysregulated miRNAs expression level in PBMCs from SS dry eye patients and healthy controls (*n* = 11 per group). D) Correlation between miR‐31‐5p expression and dry eye severity of SS dry eye patients was calculated by the Pearson correlation test. E) Real‐time qRT‐PCR analysis of miR‐31‐5p expression in LGs and PBMCs from model rabbits and normal controls (*n* = 3 per group). LG, lacrimal gland; pLGECs, purified lacrimal gland epithelial cells; PBLs: peripheral blood lymphocytes; FC: fold change; HC, healthy controls; SS, Sjogren's syndrome; BUT, tear break‐up time; CFS, corneal fluorescein staining. Data was shown as mean ± SD, and the differences were analyzed by Unpaired Student's *t*‐test. ^*^ *p* < 0.05, ^***^ *p* < 0.001.

Using global miRNA sequencing of LGs of rabbit autoimmune dacryoadenitis, we found that 6 miRNAs (miR‐381‐3p, miR‐31‐5p, miR‐656‐3p, miR‐432‐5p, miR‐34a‐5p and miR‐219a‐5p) were significantly down‐regulated at least 1.5‐fold in LGs of rabbit autoimmune dacryoadenitis compared to normal controls (**Figure** **1**A,B). Measurement of these downregulated miRNAs in PBMCs samples from SS dry eye patients showed that the levels of miR‐656‐3p, miR‐34a‐5p, and miR‐219a‐5p were all below the detection thresholds. Notably, only the expression of miR‐31‐5p was significantly lower in SS dry eye patients compared to healthy individuals, as depicted in Figure 1C. More importantly, Further correlation analysis revealed that the level of miR‐31‐5p was positively associated with tear production (*r* = 0.8347, *p* = 0.0387) and tear break‐up time (BUT) (*r* = 0.7346, *p* = 0.0242), while negatively correlated with the corneal epithelial damage scores (*r* = −0.8728, *p* = 0.0021) of SS dry eye patients (Figure 1D), suggesting the level of miR‐31‐5p is closely related to the severity of SS dry eye disease. Subsequently, this miRNA was chosen for functional studies. Then, the lower levels of miR‐31‐5p in both LGs and PBMCs of a separate cohort of rabbits with autoimmune dacryoadenitis relative to normal controls were verified by real‐time quantitative RT‐PCR (qRT‐PCR) (Figure 1E).

### 2.2 Overexpression of miR‐31‐5p Prevents the Development of Rabbit Autoimmune Dacryoadenitis

**Figure 2:**
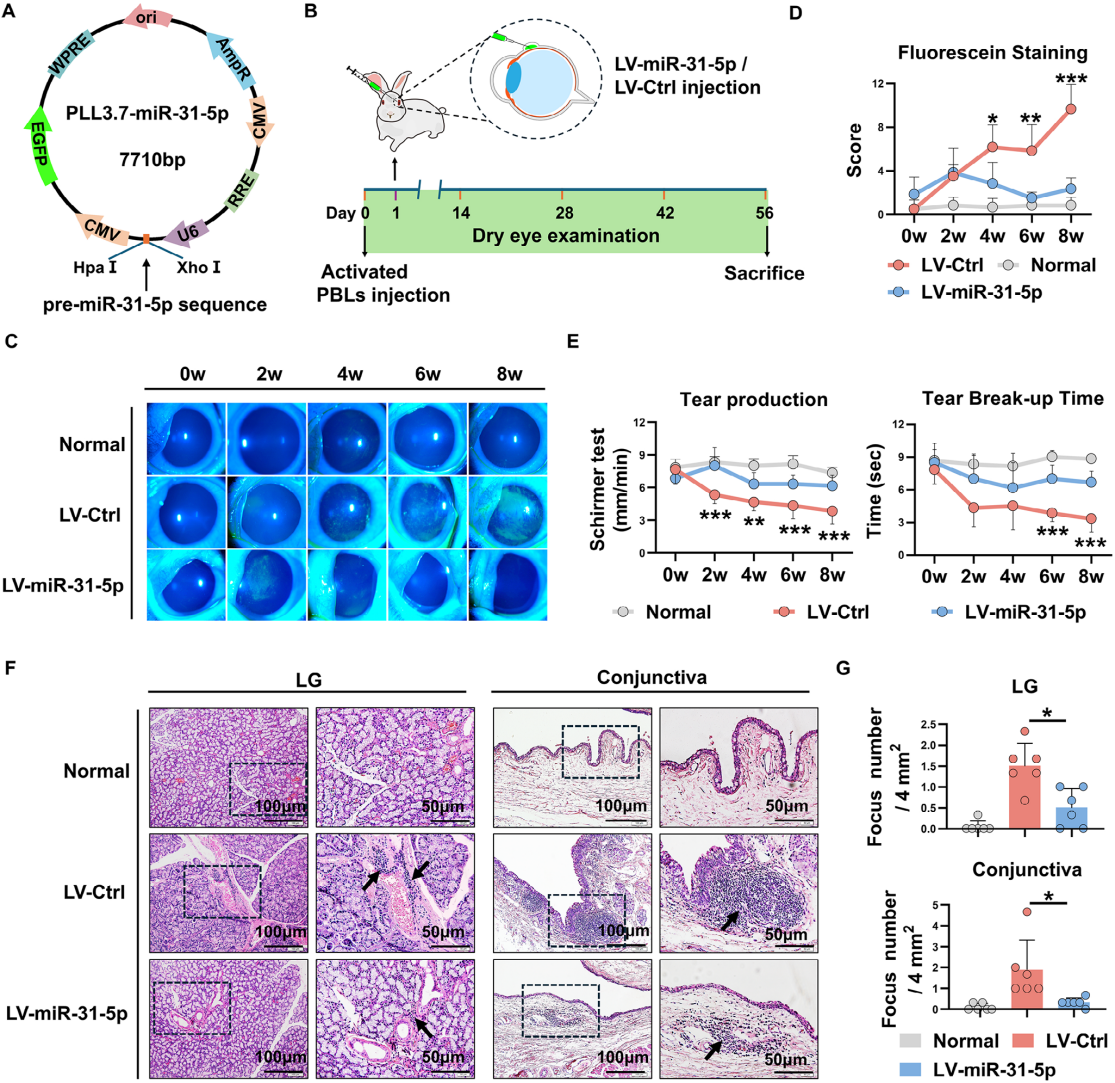
miR‐31‐5p overexpression prevents the development of rabbit autoimmune dacryoadenitis. A) Lentiviral vector plasmids used to overexpress miR‐31‐5p. B) Schematic diagram illustrating LV‐miR‐31‐5p administration at the early stage (day 1 post transfer) of rabbit autoimmune dacryoadenitis (2 × 10^7^ transducing units/eye). C) Representative corneal fluorescein staining images. D,E) Tear production, tear break‐up time and corneal fluorescein staining scores of each group of rabbits (*n* = 6/group). F,G) Representative H&E staining photographs and scores of LGs and conjunctivas in each group of rabbits (*n* = 6/group). Scale bars, 100 and 50 µm. Arrows indicate infiltrating lymphocytes. PBLs, peripheral blood lymphocytes; The data were shown as mean ± SD. ^*^ *p* < 0.05, ^**^ *p* < 0.01, ^***^ *p* < 0.001. D,E) two‐way ANOVA; G) one‐way ANOVA.

To investigate whether there is a causative link between reduced miR‐31‐5p and autoimmune dry eye, a lentiviral vector containing the pre‐miR‐31‐5p sequence was constructed, and the lentiviruses were subsequently packaged (**Figure** **2**A). Then, rabbits were subconjunctivally injected with a single dose of miR‐31‐5p‐overexpressing lentivirus at the time of disease induction (day 1 post transfer) (Figure 2B). Using real‐time qRT‐PCR, we detected significantly increased miR‐31‐5p expression in LGs of rabbits injected with miR‐31‐5p‐overexpressing lentivirus relative to the control group, indicating the successful overexpression of miR‐31‐5p in vivo (Figure S1A, Supporting Information). The severity of dry eye was evaluated by assessing tear production, BUT and corneal epithelial integrity every 2 weeks after transfer. As shown in Figure 2C–E, miR‐31‐5p overexpressing rabbits displayed significantly increased tear production, prolonged tear BUT, and milder corneal epithelial damage compared with control group rabbits. After 8 weeks of adoptive transfer, the LGs and conjunctivas were collected, and the inflammatory infiltration was assessed by H&E staining. As shown in Figure 2F,G, miR‐31‐5p overexpressing rabbits showed a significantly decreased inflammatory cell infiltration and tissue damage in the LGs and conjunctivas relative to control diseased rabbits. All together, these data suggest that miR‐31‐5p significantly attenuates the disease severity of the induced rabbit autoimmune dry eye.

### 2.3 Overexpression of miR‐31‐5p Restores the M1/M2 Macrophage Balance In Vivo

**Figure 3:**
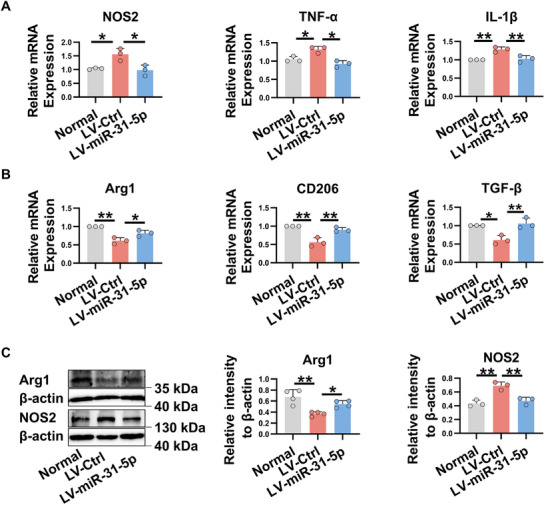
Overexpression of miR‐31‐5p restores the M1/M2 macrophage balance in vivo. LGs were collected from miR‐31‐5p overexpressing‐rabbits or control rabbits at week 8 after adoptive transfer of activated PBLs, and then subjected to real‐time qRT‐PCR or Western blot analysis. A) The relative expression of M1‐related genes. B) The mRNA expression of M2‐associated genes. C) The protein levels of Arg1 and NOS2. LV‐miR‐31‐5p, miR‐31‐5p‐overexpressing rabbits; LV‐Ctrl, control rabbits. Data was from at least three independent experiments and presented as mean ± SD. Data was analyzed by one‐way ANOVA. ^*^ *p* < 0.05, ^**^ *p* < 0.01.

Given that increased M1 macrophage activation and defective M2 macrophage polarization contribute to the development of autoimmune dacryoadenitis,^[^ ^4^, ^12^ ^]^ we next investigate the effects of miR‐31‐5p on macrophage polarization. As shown in **Figure** **3**A,B, in comparison with those in the control group, the expression of M1 macrophage marker NOS2 and M1 related pro‐inflammatory cytokines, such as TNF‐α and IL‐1β, were significantly decreased, whereas the M2 macrophage markers such as Arg1 and CD206, and anti‐inflammatory cytokines TGF‐β were dramatically increased in LGs of miR‐31‐5p‐overexpressing rabbits. Consistently, increased Arg1 and reduced NOS2 protein levels were observed in the LGs of miR‐31‐5p‐overexpressing rabbits (Figure 3C). Together, these data indicate that miR‐31‐5p can suppress M1 macrophage activation and promote M2 macrophage polarization in response to chronic inflammation in the LGs.

### 2.4 miR‐31‐5p Inhibits M1 Activation and Facilitates Macrophages into M2 Phenotype In Vitro

**Figure 4:**
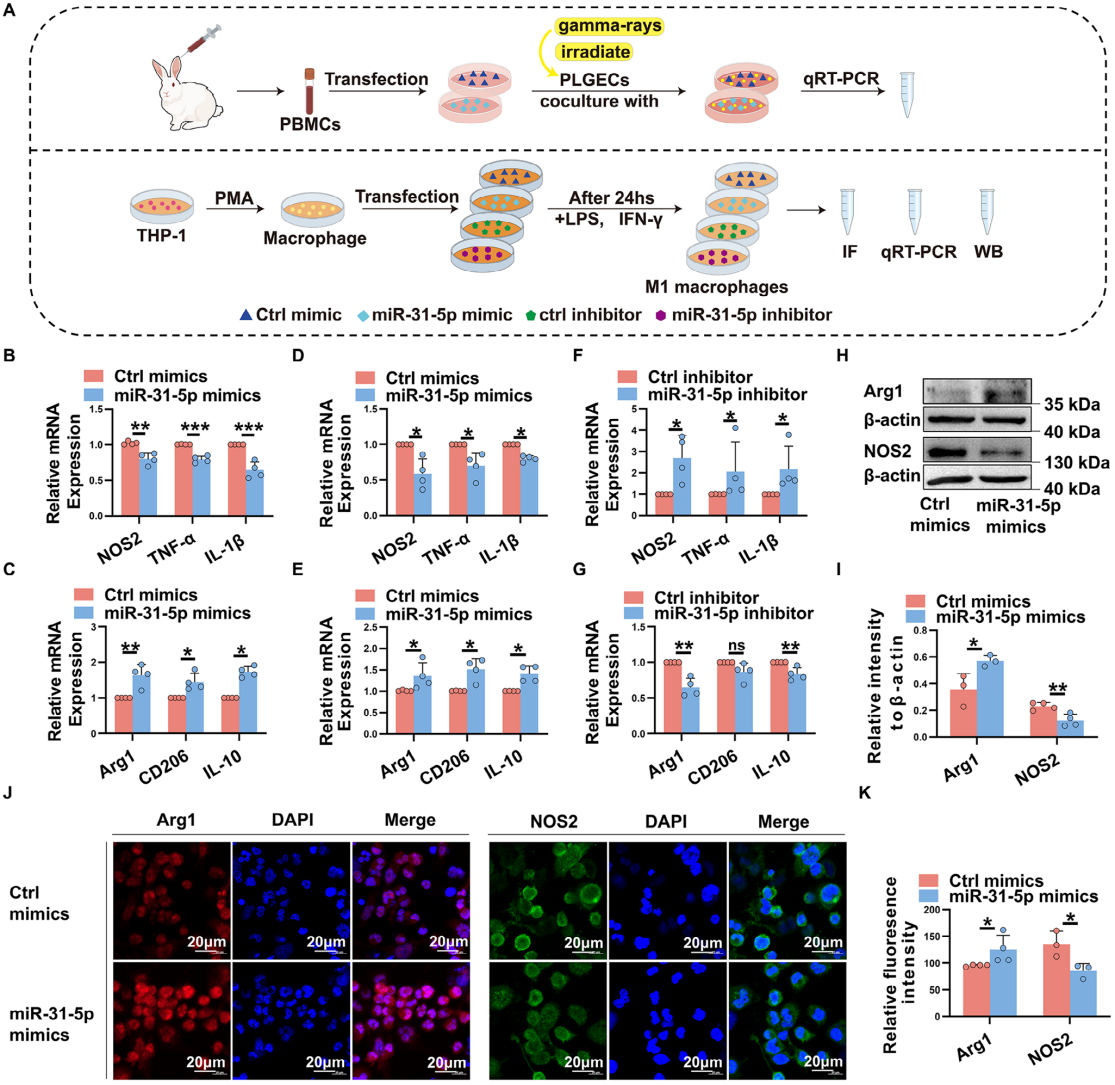
miR‐31‐5p inhibits M1 macrophage activation and facilitates macrophages into M2 phenotype in vitro. A) Scheme of experimental procedures to evaluate the role of miR‐31‐5p on macrophage polarization. B,C) PBMCs isolated from model rabbits were transfected with miR‐31‐5p mimics of control mimics (300 nmol) and cocultured with irradiated pLGECs for 48h. Relative expression of M1 markers and M2‐related genes were analyzed by real‐time qRT‐PCR. D–I) Macrophage derived from THP‐1 cells were transfected with miR‐31‐5p mimics, miR‐31‐5p inhibitors or their negative controls for 24 h, and then stimulated with LPS and IFN‐γ to induce M1 macrophage polarization. D–G) Real‐time qRT‐PCR analysis of M1 and M2 macrophage‐related gene expression. H,I) Western blot analysis of Arg1 and NOS2 protein level. J,K) Representative confocal images of Arg1 (red) and NOS2 (green) immunofluorescence staining in macrophages transfected with miR‐31‐5p mimics or control mimics. Scale bars, 20 µm. Data were representative of at least three independent experiments and were analyzed by Unpaired Student's *t*‐test or Mann–Whitney U test. ^*^ *p* < 0.05, ^**^ *p* < 0.01, ^***^ *p* < 0.001, ns, not significant.

We next investigate the effects of miR‐31‐5p on macrophage polarization in vitro. To address this, PBMCs from model rabbits were transfected with miR‐31‐5p mimics or control mimics, washed, and then stimulated with irradiated purified lacrimal gland epithelial cells (pLGECs) (**Figure** **4**A). As shown in Figure 4B,C, the mRNA levels of M1‐associated genes including NOS2, IL‐1β and TNF‐α were significantly decreased, whereas the transcript levels of M2‐related genes, such as Arg1, CD206, and IL‐10 were dramatically increased in miR‐31‐5p overexpressing group cells relative to controls.

To further confirm the macrophage‐intrinsic role of miR‐31‐5p on macrophage phenotype, THP‐1‐derived macrophages were transfected with miR‐31‐5p mimics, miR‐31‐5p inhibitor or their negative controls for 24 h, and then stimulated with LPS + IFN‐γ to induce M1 macrophage polarization (Figure 4A). real‐time qRT‐PCR analysis showed that miR‐31‐5p overexpression markedly decreased the mRNA levels of M1‐associated genes and upregulated M2‐related gene expression (Figure 4D,E), whereas blocking miR‐31‐5p had the opposite effect (Figure 4F,G). Meanwhile, significantly increased Arg1 protein expression and dramatically decreased NOS2 protein levels were observed in miR‐31‐5p mimics‐transfected macrophages (Figure 4H,I). Furthermore, immunofluorescence staining showed that the expression of M2‐related marker Arg1 was dramatically higher in miR‐31‐5p‐overexpressing macrophages relative to controls, whereas the level of M1‐associated gene NOS2 was significantly decreased in macrophages overexpressing miR‐31‐5p (Figure 4J,K). Collectively, these data indicate that miR‐31‐5p inhibits M1 macrophage activation and facilitates macrophages into an anti‐inflammatory M2 phenotype in vitro.

### 2.5 miR‐31‐5p Suppresses M1 Polarization by Targeting P2RX7

**Figure 5:**
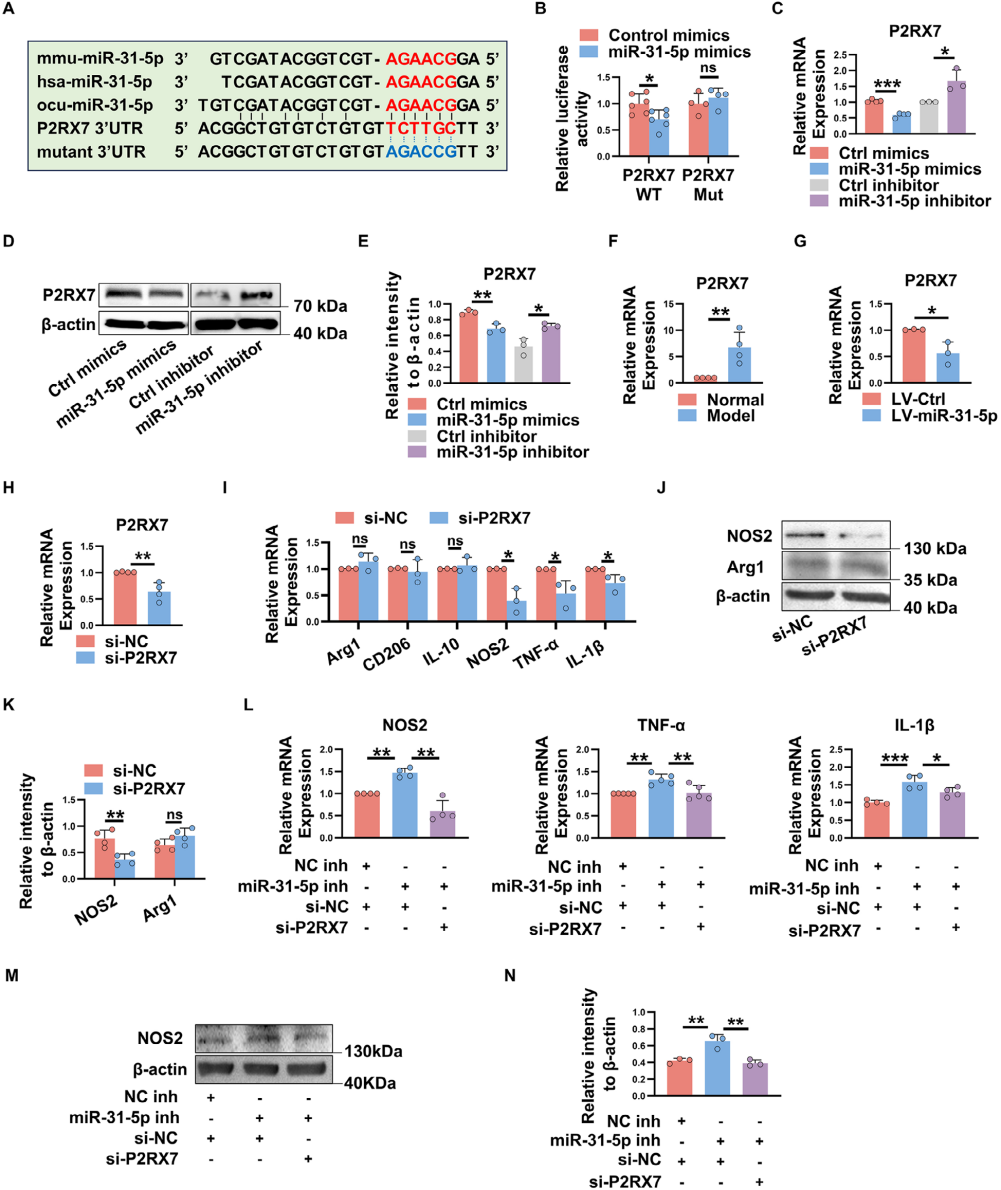
P2RX7 is a functional target of miR‐31‐5p. A) Sequence alignment between miR‐31‐5p and its potential binding sites (in red letters) in the 3^′^UTR of R2RX7 mRNA. The mutation of the miR‐31‐5p binding sites is shown in bule. B) Luciferase activity analysis of reporter carrying the wild‐type or mutant P2RX7 3^′^UTR co‐transfected into HEK293T cells with miR‐31‐5p mimics or control mimics. C–E) Macrophage derived from THP‐1 cells were transfected with indicated mimics or inhibitors, and the expression of P2RX7 was measured by real‐time qRT‐PCR and western blot. F,G) Real‐time qRT‐PCR analysis of P2RX7 expression in LGs of each group of rabbits. H–N) Macrophage derived from THP‐1 cells were transfected with indicated siRNA or inhibitors for 24 h, and then stimulated with LPS+IFN‐γ to induce M1 macrophage polarization. H) Real‐time qRT‐PCR analysis of P2RX7 expression in each group of cells. I–N) The relative expression of M1 and M2 macrophage associated genes, detected by real‐time qRT‐PCR or western blot, is shown. Data were shown as mean ± SD from at least three independent experiments. ^*^ *p* < 0.05, ^**^ *p* < 0.01, ^***^ *p* < 0.001, ns, not significant. (B‐K) Unpaired Student's I‐test; L–N) one‐way ANOVA.

To define the molecular mechanisms by which miR‐31‐5p modulates macrophage polarization, we used miRanda database to identify the potential targets of miR‐31‐5p. Further, GO category analysis focusing on immune system processes using Cytoscape software identified 15 candidate genes that were significantly enriched in immune responses (Figure S2, Supporting Information). Among these, P2RX7 garnered our attention owing to its established role in modulating macrophage‐mediated inflammation.^[^ ^30^ ^]^ As shown in **Figure** **5**A, bioinformatics analysis showed the putative miR‐31‐5p seed sequences in the 3^′^ UTR of P2RX7, and further luciferase assay showed that miR‐31‐5p suppressed the luciferase activity of a reporter with the wild‐type (WT), but not the mutant (Mut) 3^′^UTR of P2RX7 (Figure 5B), suggesting that miR‐31‐5p specifically targets P2RX7 and regulates its expression. Indeed, a gain and loss function assay showed that P2RX7 mRNA and protein levels were significantly reduced in macrophages transfected with miR‐31‐5p mimics, whereas knockdown of miR‐31‐5p dramatically increased P2RX7 expression (Figure 5C–E). In addition, we observed a significantly increased P2RX7 mRNA level in LGs of model rabbits (Figure 5F), whereas the level of P2RX7 was markedly decreased in LGs of miR‐31‐5p‐overexpressing rabbits (Figure 5G). These results indicate that P2RX7 is a functional target of miR‐31‐5p.

To determine whether P2RX7 is important for miR‐31‐5p‐mediated macrophage polarization, THP‐1‐derived macrophages were transfected with P2RX7 siRNA (Figure 5H) or control siRNA, and then stimulated with LPS + IFN‐γ. As shown in Figure 5I, the transcript levels of M1 markers were significantly decreased in P2RX7‐knockdown macrophages compared with the controls, whereas no remarkable differences were observed in the mRNA levels of M2‐related genes between the two groups. Consistently, western blot analysis showed a dramatic decrease in the protein levels of M1 marker NOS2 in P2RX7‐silenced macrophages (Figure 5J,K). Notably, knockdown of P2RX7 partially abolished the upregulation of M1‐related genes induced by miR‐31‐5p inhibitor (Figure 5L–N). These data suggest that P2RX7 is a functional target mediating reduced M1 polarization caused by miR‐31‐5p.

### 2.6 miR‐31‐5p/P2RX7 Inhibits M1 Polarization by Suppressing p38 MAPK Signaling

**Figure 6:**
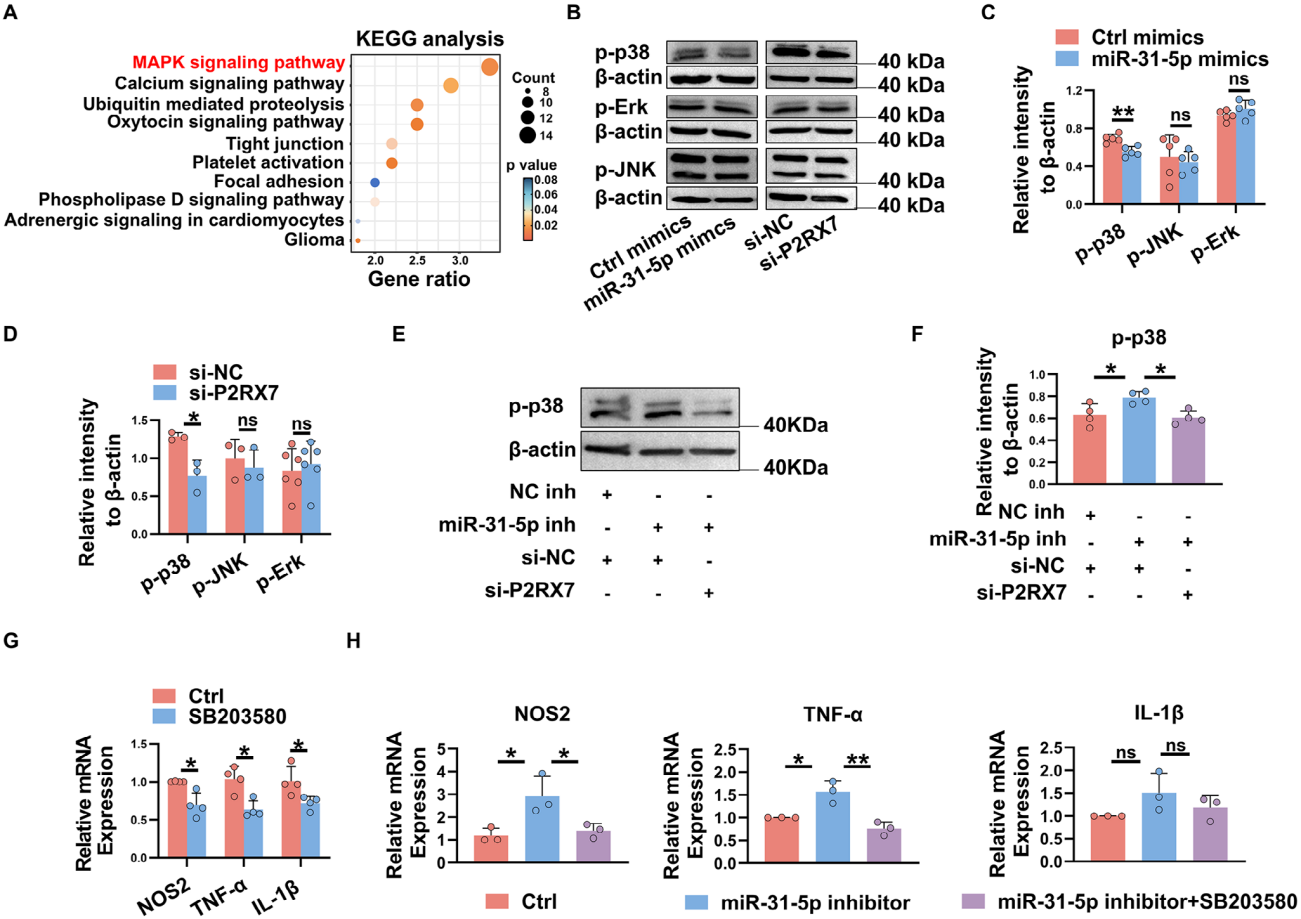
miR‐31‐5p/P2RX7 inhibits M1 macrophage polarization by suppressing p38 MAPK signaling. A) KEGG analysis on target genes of miR‐31‐5p. The top 10 pathways are summarized. B–F) THP‐1 derived macrophages were transfected with indicated mimics, siRNA or inhibitors, and then stimulated with LPS + IFN‐γ to induce M1 macrophage polarization. The phosphorylation level of p38, JNK and ERK were detected by western blot 48 h later. G) THP‐1‐derived macrophages were pretreated with 10 µm SB203580 p38 inhibitor or DMSO for 1 h prior to stimulation with LPS and IFN‐γ to induce M1 macrophage polarization. The relative mRNA expression of M1‐related genes was then examined by real‐time qRT‐PCR. I) THP‐1‐derived macrophages were transfected with miR‐31‐5p inhibitor or Ctrl inhibitor following pretreatment with 10 µm SB203580 p38 inhibitor or DMSO for 1h. The levels of M1‐associated genes were analyzed. Data were shown as mean ± SD from at least three independent experiments. ^*^ *p* < 0.05, ^**^ *p* < 0.01, ns, not significant. (C‐D and G) Unpaired Student's *t*‐test or Mann–Whitney *U* test; (F and H) one‐way ANOVA.

To elucidate the downstream pathway by which miR‐31‐5p/P2RX7 modulates M1 macrophage polarization, we performed KEGG analysis of miR‐31‐5p target gene using the DAVID database. As shown in **Figure** **6**A, the target genes of miR‐31‐5p are top enriched in MAPK pathway. Given that MAPK signaling is critical for M1 activation,^[^ ^31^ ^]^ and that P2RX7 has been reported to activate the MAPK pathway in Osteoclasts,^[^ ^32^ ^]^ we next examined whether the MAPK pathway is involved in the action of miR‐31‐5p on macrophage polarization. To this end, macrophages transfected with miR‐31‐5p mimics, P2RX7 siRNA, or their negative controls were stimulated to polarize toward M1 macrophages, and the phosphorylation levels of p38, JNK and ERK were monitored 48 h later. As shown in Figure 6B–D, both miR‐31‐5p overexpression and P2RX7 knockdown dramatically reduced the phosphorylation levels of p38, while the phosphorylation level of JNK and ERK showed no difference between the two groups. Notably, silencing P2RX7 partially reversed the increased levels of p‐p38 induced by miR‐31‐5p inhibitor (Figure 6E,F). These data suggest that miR‐31‐5p/P2RX7 might suppress M1 macrophage polarization by regulating the p38 MAPK pathway.

To further investigate the role of p38 MAPK pathway in modulating macrophage polarization, we pretreated THP1‐derived macrophages with the p38 inhibitor SB203580 for 1 h prior to stimulating them with LPS and IFN‐γ to induce M1 macrophage polarization, and we found that inhibiting the p38 MAPK signaling pathway significantly decreased the transcript levels of M1 markers, including NOS2, IL‐1β and TNF‐α (Figure 6G), suggesting the important role of the p38 MAPK pathway in modulating the function and phenotype of macrophages. Notably, inhibition of p38 MAPK signaling partially reversed the upregulation of M1‐related genes induced by the miR‐31‐5p inhibitor (Figure 6H), indicating that the p38 MAPK pathway is closely involved in miR‐31‐5p‐mediated macrophage polarization.

### 2.7 miR‐31‐5p is Regulated by m6A Methylation

**Figure 7:**
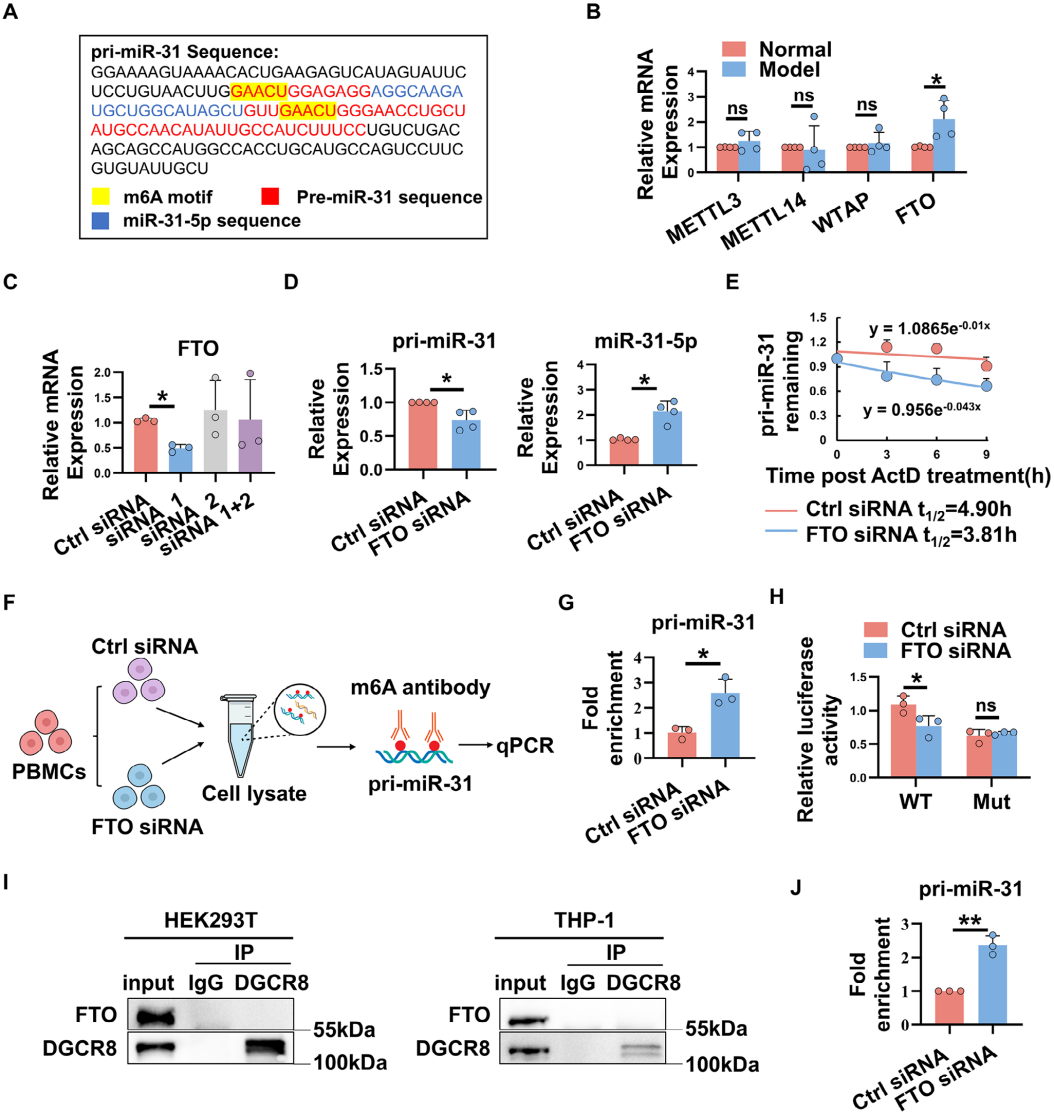
m6A methylation regulates the expression of miR‐31‐5p in autoimmune dry eye. A) The sequences of pri‐miR‐31, miR‐31‐5p, and the potential m6A motif (GAACU) were highlighted with different colors. B) Relative expression of m6A enzymes (METTL3, METTL14, WTAP and FTO) in LGs of normal and model rabbits were detected by real‐time qRT‐PCR. C,D) PBMCs from model rabbits were transfected with indicated FTO siRNA. The relative expression of FTO, pri‐miR‐31‐5p and miR‐31‐5p was detected by real‐time qRT‐PCR. E) Pri‐miR‐31 levels in FTO knockdown and negative control PBMCs after actinomycin D treatment at the indicated times. F) Flow diagram of MeRIP‐qPCR assays. G) MeRIP‐qPCR analysis of the m6A levels of pri‐miR‐31 in FTO silenced PBMCs and control cells. H) Luciferase activity analysis was performed on a reporter carrying the wild‐type or mutant sequence of pri‐miR‐31, which was co‐transfected into HEK293T cells with either FTO siRNA or control siRNA. I) Total protein from HEK293T and THP‐1 cells was immunoprecipitated with an anti‐DGCR8 antibody. Western blots for FTO and DGCR8 are shown. J) Detection of the abundance of pri‐miR‐31 binding to DGCR8 in HEK293T cells by immunoprecipitation with an anti‐DGCR8 antibody. Data were shown as mean ± SD, and the differences were analyzed by one‐way ANOVA, Unpaired Student's *t*‐test or Mann–Whitney *U* test. ^*^ *p* < 0.05, ^**^ *p* < 0.01, ns, not significant.

m6A, the most prevalent modification of mRNAs and noncoding RNAs, plays an important role in miRNA biogenesis through modulating pri‐miRNA processing and miRNA maturation.^[^ ^33^ ^]^ The m6A modification mainly occurs at the consensus motif “RRACH” (R = G or A; H = A, C or U).^[^ ^34^ ^]^ To explore why miR‐31‐5p is decreased in autoimmune dry eye, we first sought for the m6A sites on pri‐miR‐31 sequence using SRAMP. As shown in **Figure** **7**A, we found two potential m6A sites located near the splicing site of pri‐miR‐31, suggesting that m6A may play a role in the regulation of miR‐31‐5p during dry eye.

To determine m6A enzymes involved in the regulation of miR‐31‐5p expression in autoimmune dry eye, major m6A writers (METTL3, METTL14, and WTAP) and erasers (FTO) expression levels in LGs of model rabbits and normal controls were evaluated by real‐time qRT‐PCR. As shown in Figure 7B, the m6A demethylase FTO, but not other enzymes, was significantly elevated in LGs of model rabbits, suggesting that FTO may be involved in the dysregulation of miR‐31‐5p in autoimmune dry eye. Indeed, using siRNA‐mediated knockdown of FTO in PBMCs from model rabbits (Figure 7C), we found that silencing FTO significantly decreased pri‐miR‐31 expression, whereas miR‐31‐5p levels were dramatically upregulated (Figure 7D), indicating that FTO may regulate the processing of miR‐31‐5p via m6A. This was further confirmed by the observation that depletion of FTO dramatically decreased the half‐life of pri‐miR‐31 in PBMCs from model rabbits (Figure 7E). Importantly, Methylated RNA immunoprecipitation qPCR (MeRIP‐qPCR) analysis uncovered that m6A was highly enriched within the pri‐miR‐31 sequence, and the enrichment m6A level was significantly upregulated following the knockdown of FTO (Figure 7F,G). To further validate the impact of FTO on pri‐miR‐31 processing through m6A, we constructed dual‐luciferase reporters containing WT (containing potential m6A sites) or Mut forms (the adenosine base in m6A consensus sequences replaced with thymine to abolish the m6A modification) of pri‐miR‐31. As shown in Figure 7H, FTO knockdown significantly decreased the luciferase activity in the WT group but not in the Mut group, further supporting the m6A modification in pri‐miR‐31 processing. The m6A modification has been proved to facilitate the recognition of pri‐miRNA hairpin by DiGeorge syndrome critical region 8 (DGCR8), thus promoting miRNAs maturation.^[^ ^19^ ^]^ We next asked whether FTO regulated pri‐miR‐31 processing in a DGCR8‐dependent manner. To this end, total protein from HEK293T and THP‐1 cells, where both FTO and DGCR8 were expressed, was immunoprecipitated with an anti‐DGCR8 antibody. As presented in Figure 7I, we found that FTO was not co‐precipitated with DGCR8 in either HEK293T or THP‐1 cell lines, indicating that FTO did not directly interact with DGCR8. Further RNA immunoprecipitation (RIP) experiments showed that knockdown of FTO significantly increased the enrichment of pri‐miR‐31 in the complex precipitated with antibody against DGCR8 (Figure 7J), suggesting that FTO modulates miR‐31‐5p processing by influencing the recognition of pri‐miR‐31 by DGCR8. Together, these data indicate that FTO mediated m6A demethylation, which sustained pri‐miR‐31 stability, was responsible for decreased miR‐31‐5p.

### 2.8 Overexpression of miR‐31‐5p at the Developed Stage Efficiently Alleviates Autoimmune Dacryoadenitis in Rabbits

**Figure 8:**
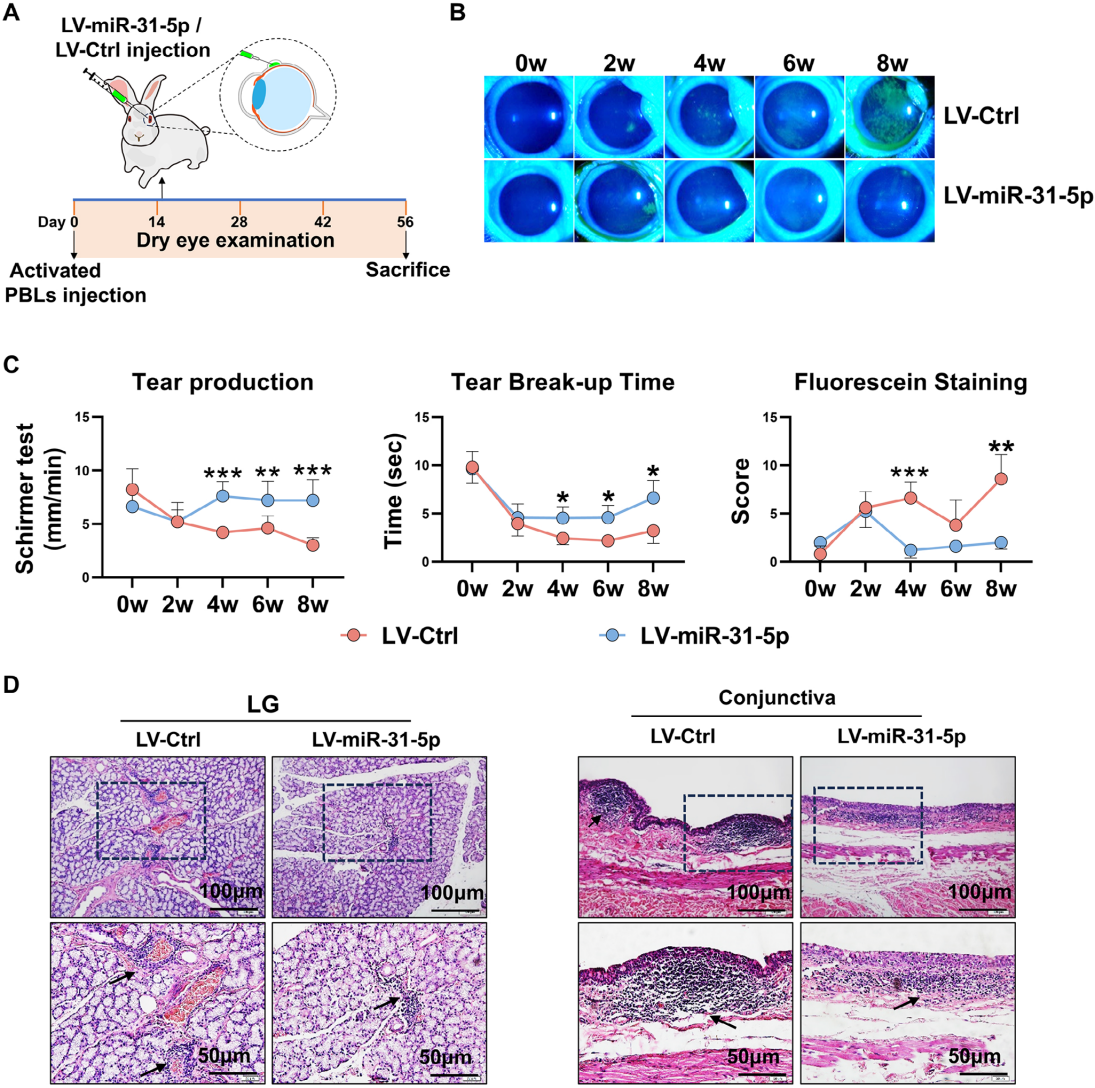
Overexpression of miR‐31‐5p at the developed stage efficiently alleviates autoimmune dacryoadenitis in Rabbits. A) Schematic diagram illustrating LV‐miR‐31‐5p administration (2 × 10^7^ transducing units/eye) at the developed stage (day 15 post transfer) of rabbit autoimmune dacryoadenitis. B) Representative corneal fluorescein staining images. C) Tear production, tear break‐up time and corneal fluorescein staining scores of each group of rabbits (n = 5/group). D) Representative specimens of H&E staining in LGs and conjunctivas. Scale bars, 100 and 50 µm. Arrows indicate infiltrating lymphocytes. PBLs, peripheral blood lymphocytes. Data was shown as mean ± SD and analyzed by two‐way ANOVA. ^*^ *p* < 0.05, ^**^ *p* < 0.01, ^***^ *p* < 0.001.

The induced rabbit autoimmune dacryoadenitis typically exhibits evident clinical dry eye symptoms starting from the 2 weeks following the adoptive transfer of activated peripheral blood lymphocytes (PBLs).^[^ ^4^, ^35^ ^]^ To determine whether overexpression of miR‐31‐5p had therapeutic effects on rabbit autoimmune dacryoadenitis, a single dose of miR‐31‐5p‐overexpressing lentivirus was injected subconjunctivally into rabbits after disease onset (day 15 after transfer), as illustrated in **Figure** **8**A. Clinically, miR‐31‐5p overexpression (Figure S1B, Supporting Information) significantly alleviated dry eye symptoms, as evidenced by increased tear secretion, prolonged tear BUT, and decreased corneal fluorescein staining (CFS) scores compared to the control group (Figure 8B,C). Histologically, the infiltration of inflammatory cells in LGs and conjunctivas was significantly attenuated in miR‐31‐5p‐overexpressing rabbits (Figure 8D). Together, the administration of miR‐31‐5p‐overexpressing lentivirus effectively attenuated the severity of autoimmune dry eye in rabbits at the developed stage of the disease.

### 2.9 Downregulation of miR‐31‐5p is Associated with Increased M1 Macrophage Activation in SS Dry Eye Patients

**Figure 9:**
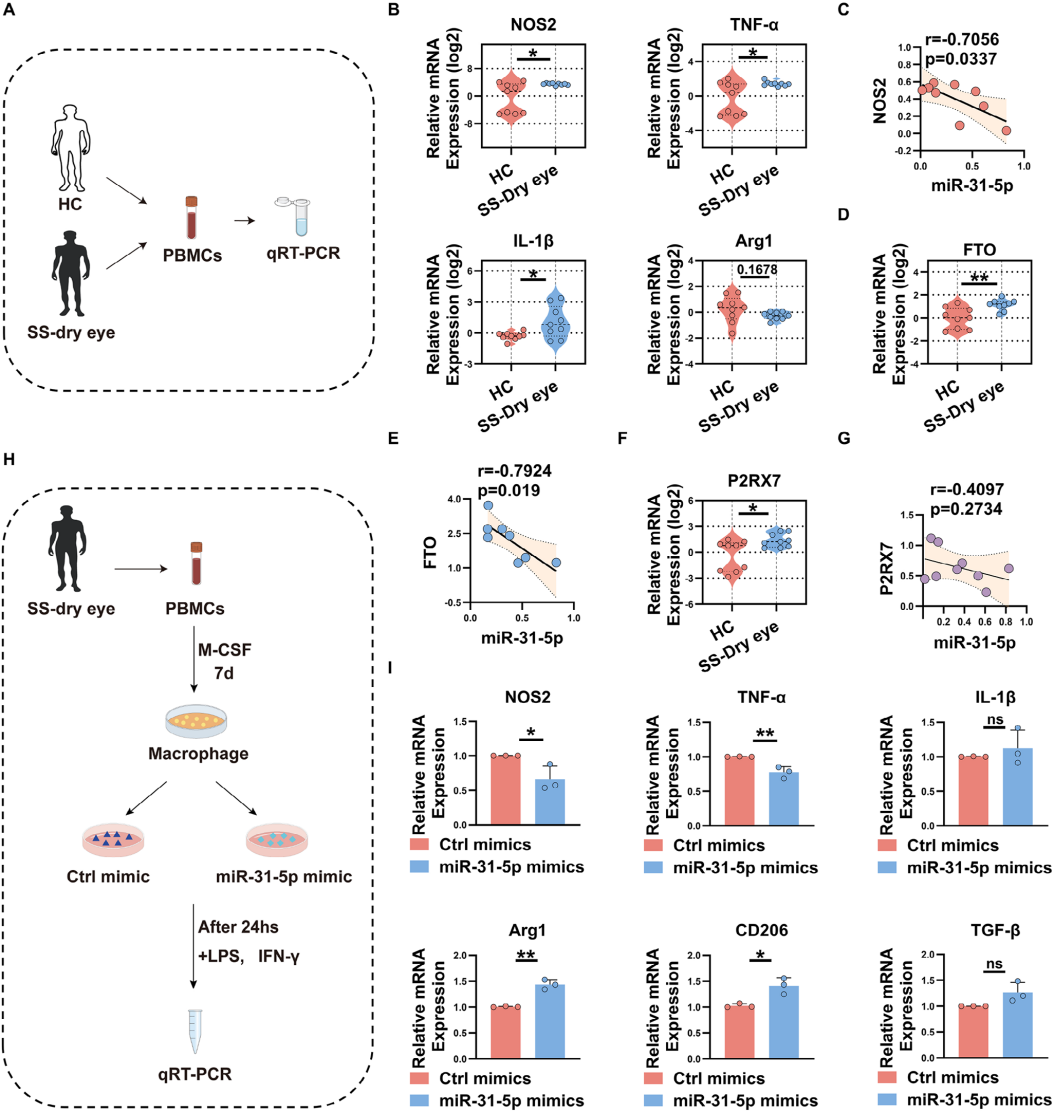
Downregulation of miR‐31‐5p is associated with increased M1 macrophage activation in SS dry eye patients. A) Schematic outline of experimental procedures designed to assess gene expression in patients with SS dry eye patients. B) Expression levels of macrophage‐related genes in PBMCs from SS dry eye patients and controls subjects. C) Correlation analysis between miR‐31‐5p expression and NOS2 levels in PBMCs of SS dry eye patients. D,E) Quantification of FTO mRNA levels in PBMCs of SS dry eye patients and its correlation with miR‐31‐5p expression. F,G) Expression of P2RX7 in PBMCs of SS dry eye patients and its association with miR‐31‐5p level. H,I) PBMC‐derived macrophages from SS dry eye patients were transfected with miR‐31‐5p mimics or negative controls and subsequently polarized into M1 macrophages. The expression of M1 and M2 macrophage‐related gene was measured by real‐time qRT‐PCR. Data was shown as mean ± SD and analyzed by Unpaired Student's *t*‐test or Mann–Whitney U test. ^*^ *p* < 0.05, ^**^ *p* < 0.01.

As demonstrated by the results obtained both in vivo and in vitro, miR‐31‐5p is implicated in the pathogenesis of autoimmune dry eye by facilitating M1 macrophage polarization via targeting P2RX7, we next aim to validate these results using samples from SS dry eye patients. To this end, PBMCs isolated from both healthy controls and SS dry eye patients were collected and analyzed (**Figure** **9**A). As shown in Figure 9B, we first noted a significant upregulation of M1‐related genes, including NOS2, TNF‐α, and IL‐1β, in SS dry eye patients, whereas no statistically significant differences were observed in the expression of M2 markers, such as Arg1, pointing to M1 macrophage activation in the progression of SS dry eye. Specifically, we discovered that the decreased miR‐31‐5p level (Figure 1C) significantly correlated with higher NOS2 expression (*r* = −0.7056, *p* = 0.0337) in SS dry eye patients (Figure 9C). These data indicated that reduced miR‐31‐5p may contribute to M1 macrophage activation and autoinflammation in autoimmune dry eye. Additionally, we observed a dramatic increase in FTO expression in SS dry eye patients, as shown in Figure 9D. Based on our in vitro data, the upregulation of FTO may impede the maturation of miR‐31‐5p and reduce its expression, thereby dampening the inhibitory effect of miR‐31‐5p on its target gene, P2RX7. Of note, a negative correlation was observed between FTO and miR‐31‐5p levels (*r* = −0.7924, *p* = 0.019) in SS dry eye patients (Figure 9E). Furthermore, SS dry eye patients exhibited a markedly elevated P2RX7 level, which displayed a negative trend, albeit not statistically significant, in correlation with miR‐31‐5p (*r* = −0.4097, *p* = 0.2734) (Figure 9F,G). Together, these data support the existence of potential crosstalk between the FTO‐miR‐31‐5p‐P2RX7 axis and M1 macrophage activation in human autoimmune dry eye.

To further investigate the clinical significance of miR‐31‐5p, PBMC‐derived macrophages from SS dry eye patients were transfected with miR‐31‐5p mimics or negative controls and subsequently polarized into M1 macrophages (Figure 9H). As shown in 9I, the overexpression of miR‐31‐5p significantly reduced the mRNA expression of M1‐related genes (NOS2, TNF‐α) while upregulating the levels of M2‐associated genes (Arg1, CD206). Taken together, our data strongly suggest that miR‐31‐5p is involved in modulating macrophage polarization in SS dry eye patients.

## 3 Discussion

**Figure 10:**
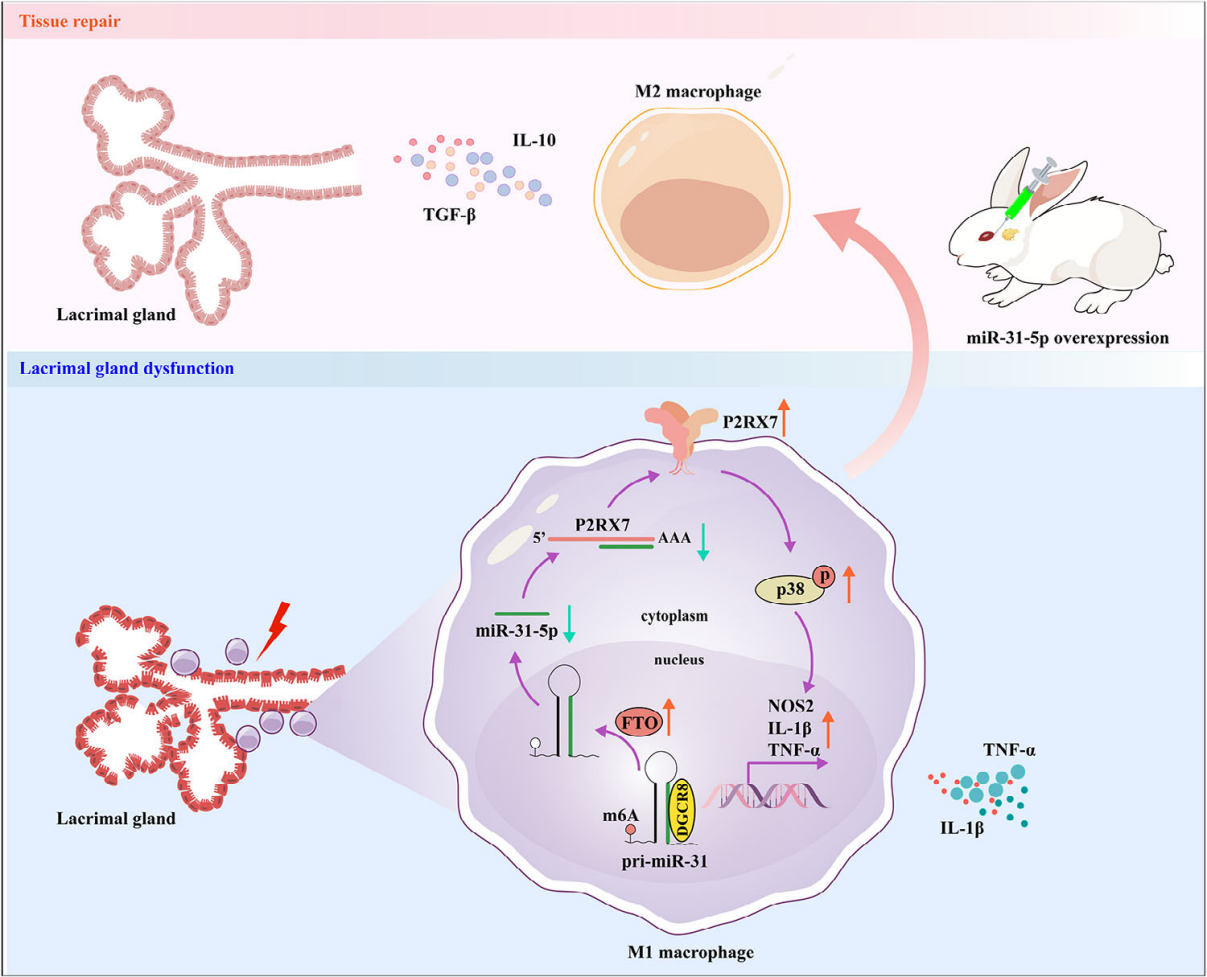
Schematic depicting the mechanisms by which miR‐31‐5p regulates M1/M2 balance in autoimmune dry eye. The upregulation of FTO in autoimmune dry eye suppresses the recognition of pri‐miR‐31 by DGCR8, thereby inhibiting the maturation of miR‐31‐5p and reducing its inhibitory effect on the target gene P2RX7. The activation of P2RX7 subsequently triggers the p38 MAPK signaling pathway and increases the level of M1‐related genes, thus exacerbating the development of autoimmune dry eye. Conversely, overexpression of miR‐31‐5p converts M1 macrophages into an M2 phenotype, which secretes anti‐inflammatory mediators and alleviates the symptoms of autoimmune dry eye.

Through miRNA sequencing of LGs from model rabbits, together with validation in PBMCs of SS dry eye patient, we characterized miR‐31‐5p as a potential immunoregulator of autoimmune dry eye. Importantly, using the rabbit autoimmune dacryoadenitis model, we demonstrated that miR‐31‐5p can protect rabbits against autoimmune dacryoadenitis by modulating M1/M2 balance through suppressing P2RX7 and inactivating p38 MAPK signaling (**Figure** **10**).

Previous studies, including our own, have demonstrated the pivotal role of M1/M2 macrophages in regulating autoimmune dry eye, among which M1 macrophages are closely involved in the progression of autoimmune dry eye lesions, while M2 is critical for resolving chronic inflammation and slowing down disease progression.^[^ ^4^, ^12^ ^]^ miR‐31‐5p has been linked to macrophage function. In RAW264.7 macrophages, overexpression of miR‐31‐5p obviously downregulated the expression of LPS‐induced inflammatory factors including TNF‐α, IL‐6, and IL‐1β.^[^ ^36^ ^]^ Nonetheless, little is known about the role of miR‐31‐5p in macrophage polarization. In this study, we for the first time reported that overexpression of miR‐31‐5p dramatically augmented the expression of M2 markers (Arg1 and CD206), while it markedly decreased M1 marker NOS2 expression both in vivo and in vitro, suggesting a critical role of miR‐31‐5p in modulating M1/M2 balance. In addition, using THP‐1‐derived macrophages, we further demonstrated that miR‐31‐5p possessed the ability to shift M1 macrophage to an anti‐inflammatory M2 phenotype, underscoring the pivotal role of macrophage‐intrinsic miR‐31‐5p in regulating macrophage phenotypes. Increased M2 macrophages induced by miR‐31‐5p may result in the attenuated autoimmune dacryoadenitis observed in miR‐31‐5p overexpressing model rabbits. Considering that M1 activation in inflamed LGs induced tissue destruction in autoimmune dry eye, targeting miR‐31‐5p may be a promising therapeutic strategy.

P2RX7, a ligand‐gated ion channel that is abundantly expressed in macrophages, plays a pivotal role in the initiation and progression of inflammatory and autoimmune disorders.^[^ ^37^ ^]^ Here, utilizing the miRanda database and luciferase reporter assays, we identified P2RX7, which has been documented to be upregulated in PBMCs of SS dry eye patients,^[^ ^38^ ^]^ was a novel target of miR‐31‐5p in macrophages. Using LPS/IFN‐γ‐induced M1 macrophages, we found that knockdown of P2RX7 significantly reduced the expression of M1‐related genes in vitro. This finding is further supported by a previous report showing that P2RX7 drives the secretion of M1 macrophage cytokines in human monocyte‐derived macrophages.^[^ ^39^ ^]^ Importantly, we demonstrated that the knockdown of P2RX7 dramatically rescued the increased M1 markers in macrophages induced by miR‐31‐5p knockdown, indicating an important regulatory role of P2RX7 in miR‐31‐5p mediated macrophage polarization in autoimmune dry eye. Nonetheless, the knockdown of P2RX7 had no obvious effect on M2‐related genes. This suggests that miR‐31‐5p may modulate macrophage polarization through other additional mechanisms beyond targeting P2RX7, which need further exploration. miR‐31 has been proven to exert pro‐inflammatory or immune‐suppressive effects in various autoimmune disorders by interacting with a broad spectrum of molecular targets in specific tissues and cellular contexts. Through targeting signal transducer and activator of transcription 1 (STAT1)^[^ ^40^ ^]^ and carcinoembryonic antigen‐related cell adhesion molecule 1 (CEACAM1),^[^ ^41^ ^]^ miR‐31 has been found to blunt Th1 responses and enhance Treg cell differentiation, thereby exerting a protective effect during HIV infection and the progression of systemic lupus erythematosus. Conversely, by suppressing SH2 domain containing 1A (SH2D1A)^[^ ^42^ ^]^ and GPRC5A,^[^ ^16^ ^]^ miR‐31 promotes Th1 cytokine transcription but suppresses Treg generation, thus promoting autoimmunity in sepsis and experimental autoimmune encephalomyelitis. Our findings here uncover a previously unrecognized functional interplay between miR‐31‐5p and P2RX7 in macrophage polarization, which adds another layer to the complicated regulatory network of miR‐31‐5p in autoimmunity.

MAPKs, including p38, ERK, and JNK, are a group of highly conserved serine/threonine protein kinases in eukaryotes, orchestrating various intracellular activities such as inflammation and innate immunity.^[^ ^43^, ^44^ ^]^ P2RX7 has been linked to MAPK pathways in an intranigral lipopolysaccharide rat model of Parkinson's disease^[^ ^45^ ^]^ and in septic mice,^[^ ^46^ ^]^ but according to bioinformatic analysis and validation through in vitro experiments, we are the first to find the interaction between P2RX7 and MAPK signaling in macrophage polarization. We found that silencing P2RX7 selectively inhibited the activation of p38, but not ERK and JNK, in macrophages. Previous studies have documented the crucial role of p38 MAPK signaling in the transcriptional activation of M1 macrophage‐related genes.^[^ ^47^, ^48^ ^]^ In line with these observations, we found that inhibiting p38 MAPK activity using SB203580 led to a substantial decrease in the transcript levels of M1 marker NOS2, as well as the polarizing cytokines IL‐1β and TNF‐α. Importantly, we demonstrated that blocking p38 MAPK antagonized increased M1 related molecules caused by silencing miR‐31‐5p, and knockdown of P2RX7 rescued the increased phosphorylation level of p38 MAPK induced by the miR‐31‐5p inhibitor. These results suggested that the P2RX7‐p38 axis is an important downstream mechanism involved in the action of miR‐31‐5p on M1 activation.

m6A is a critical post‐transcriptional regulator that can modulate pri‐miRNA processing and miRNA maturation, thereby functioning in a variety of diseases such as cancer and autoimmune disorders.^[^ ^25^, ^26^, ^49^ ^]^ Our previous research has uncovered that METTL3‐induced miR‐338‐3p promoted pathogenic Th17 cell responses and retinal inflammation in EAU.^[^ ^26^ ^]^ By altering the m6A modification status of pri‐miRNA, FTO has been reported to block the maturation of miR‐17‐5p in triple‐negative breast cancer (TNBC) cells and decrease miR‐3591‐5p expression in chondrocytes, thereby suppressing the progression of TNBC and osteoarthritis.^[^ ^27^, ^50^ ^]^ However, the functions of m6A modified miRNAs in autoimmune dry eye remain elusive. In this study, we identified the m6A modification at the pri‐miR‐31 sequence by a series of experiments. Importantly, using MeRIP‐qPCR, Luciferase reporter assay, and RNA stability analysis, we uncovered that knockdown of FTO dramatically upregulated the m6A level on pri‐miR‐31 transcripts and shortened their half‐life, indicating that FTO‐mediated m6A demethylation may maintain the stability of pri‐miR‐31, thereby reducing its expression in autoimmune dry eye. RNA‐binding protein DGCR8 plays an important role in miRNA maturation. FTO has been reported to suppress the binding of DGCR8 to pri‐miRNA through m6A demethylation, thus restraining miR‐138‐5p and miR‐3591‐5p maturation.^[^ ^27^, ^51^ ^]^ Consistent with these findings, we also observed that knockdown of FTO significantly increased the interaction between pri‐miR‐31 and DGCR8, suggesting that FTO‐mediated m6A demethylation affects the recognition of pri‐miR‐31 by DGCR8, thereby inhibiting the maturation of miR‐31‐5p. However, contrasting evidence also exists. For example, m6A modification on pri‐miRNA‐374c has been shown to impede the maturation of miRNA‐374c‐5p in T‐47D cells.^[^ ^52^ ^]^ Different cell types and disease contexts may explain the discrepancy among these studies.

LGs samples of SS dry eye patients are very difficult to obtain. To provide an alternative clinical link of our findings with SS dry eye, we have measured the levels of miR‐31‐5p in PBMCs from SS dry eye patients, and we found that levels of miR‐31‐5p were significantly lower in SS dry eye patients than those in healthy controls, which were consistent with miR‐31‐5p downregulation in LGs of rabbit dry eye models. Furthermore, we observed that miR‐31‐5p not only significantly reduced the expression of M1‐related markers while upregulating the levels of M2‐associated genes in PBMCs from SS dry eye patients, but also were closely associated with clinical parameters of SS dry eye patients, suggesting the clinical importance of miR‐31‐5p. Thus, miR‐31‐5p in PBMCs may be a novel biomarker for SS dry eye patients.

Despite the promising findings, this study is limited by its focus on examining the role of miR‐31‐5p in the macrophages. It remains to be determined whether and how miR‐31‐5p affects other immune cells that also play important roles in autoimmune dry eye, such as CD4^+^ T cells. Moreover, miRNAs can target many gene transcripts, so our study may miss some related targets that are also involved in the action of miR‐31‐5p. Furthermore, studies involving more clinical samples, including PBMCs, tears, and serum from patients at different disease stages and with different treatment responses, would be of great importance to prove the relationship between miR‐31‐5p and disease characteristics in depth. In addition, we first paid attention to the downregulated miRNAs in this study. Given that both upregulated and downregulated miRNAs may contribute to the development of autoimmune dry eye disease, more studies are needed to investigate the role of those significantly upregulated miRNAs in SS dry eye.

In summary, our data demonstrated that m6A modified miR‐31‐5p can act by selectively suppressing P2RX7‐p38 pathway to restore M1/M2 balance, thereby alleviating autoimmune dry eye. These findings shed new light on autoimmune dry eye mechanisms and may provide a promising therapeutic target for the treatment of autoimmune dry eye.

## 4 Experimental Section

### Animals

One‐year‐old female New Zealand white rabbits, weighing between 3.5 and 4 kg, were purchased from Vital River Laboratory Animal Technology (Beijing, China). All rabbits were housed in a pathogen‐free environment at Tianjin Medical University Eye Hospital, where the temperature was controlled at 25 °C ± 2 °C, the relative humidity ranged from 50% to 75%, and a 12‐h light‐dark cycle (from 8 am to 8 pm) was maintained. The research adhered strictly to the guidelines of the ARVO Statement for the Use of Animals in Ophthalmic and Vision Research, and the experimental procedures were approved by the Institutional Animal Care and Use Committee of Tianjin Medical University Eye Hospital (Permit Number: TJYY2023120210).

### Human Samples

PBMC samples from eleven SS dry eye patients (female; mean age, 57.5±13.5 years) and eleven age‐matched healthy controls (female; mean age, 55.7±7.9 years) were collected from Tianjin Medical University Eye Hospital biobank and used to evaluate gene expression. All SS dry eye patients were diagnosed based on the American College of Rheumatology/European League Against Rheumatism (ACR/EULAR) 2016 criteria. The study adhered to the Helsinki Declaration and was approved by the Ethics Committee of Tianjin Medical University Eye Hospital (Ethical batch number: 2021KY‐17). Written informed consent was obtained from all participants. Dry eye examination was conducted for SS dry eye patients, including measurement of tear BUT, Schirmer I test, and assessment of CFS scores.

### Rabbit Autoimmune Dacryoadenitis Induction and Evaluation

Autoimmune dacryoadenitis was induced in rabbits using the protocol described by Wei et al.^[^ ^53^ ^]^ Briefly, left inferior LGs and peripheral blood were harvested from each rabbit for isolating pLGECs and PBLs, respectively. After two days of separate cultivation, the pLGECs were irradiated (25 Gy) and subsequently cocultured with an equal number of PBLs (1 × 10^6^/well) for a duration of 5 days. Autoimmune dacryoadenitis was induced by injecting the activated lymphocytes (2 × 10^6^ cells) back into rabbits through ear margin veins.

To monitor the progression of autoimmune dacryoadenitis following the injection of activated PBLs, dry eye clinical evaluations were conducted biweekly, encompassing assessments of tear production, tear BUT, and CFS scores. Histopathological evaluation of LGs and conjunctivas was performed by hematoxylin and eosin (H&E) staining at the end of the experiment. In brief, the rabbits were sacrificed and the left eyeball and the right inferior LGs were surgically removed. Then the eyeball and half of the LGs were fixed in 10% formalin, embedded in paraffin, routinely stained with H&E, and then photographed using a BX51 microscope (Olympus Corporation, Tokyo, Japan). The number of focus (aggregates of >50 lymphocytes) per 4 mm^2^ in LGs and conjunctivas was recorded by two experienced technicians in a blinded manner.

### Plasmid Construction, Lentivirus Preparation and Administration

miRNAs originate from pri‐miRNAs in the nucleus, which are then cleaved into pre‐miRNAs. These pre‐miRNAs are subsequently exported to the cytoplasm, where they undergo final processing into mature miRNAs.^[^ ^54^ ^]^ To construct a lentiviral vector (LV‐miR‐31‐5p) that overexpresses miR‐31‐5p, an 86 bp fragment containing the rabbit pre‐miR‐31‐5p sequence was amplified and cloned into the PLL3.7 plasmid (Addgene, Watertown, USA) using Hpa I and Xho I restriction enzymes (Takara, Kusatsu, Japan). The recombinant plasmid was verified by sequencing and the lentivirus was produced according to the manufacturer's instructions. For administration in rabbits, a single dose of lentiviruses (2 × 10^7^ transducing units) was injected subconjunctivally following the adoptive transfer of activated PBLs (day 1) or after the onset of disease (2 weeks after transfer). The following sequences of primers for pre‐miR‐31‐5p amplification were used: forward primer 5^′^‐TGTGGAGAGGAGGCAAGATGCTGGC‐3^′^ and reverse primer 5^′^‐ CCGCTCGAGAAAAAAAAGATGGCAATATGTTGGC‐3^′^.

### THP‐1 Cell Culture and PBMC Derived Macrophages Differentiation

The Human monocytic THP‐1 cell line (Stem Cell Bank, Chinese Academy of Sciences) was cultured in Gibco RPMI 1640 medium supplemented with 10% FBS (Gibco, USA), 1% Penicillin/Streptomycin (Gibco, USA), and 45 µm β‐mercaptoethanol (Gibco, USA) at 37 °C in a 5% CO_2_ incubator. To generate M0 macrophages, 1 × 10^6^ ml^−1^ THP‐1 cells (third to seventh passage) were seeded in a 24‐well plate and treated with 160 ng ml^−1^ PMA (Sigma–Aldrich, Saint Louis, MO, USA) for 12h. Subsequently, the PMA‐stimulated THP‐1 cells were further stimulated with 100 ng ml^−1^ LPS and 50 ng ml^−1^ IFN‐γ (R&D Systems, Minneapolis, MN, USA) for an additional 48 h to obtain M1 macrophages.

PBMCs were isolated from freshly obtained peripheral blood of SS dry eye patients using gradient centrifugation techniques. These PBMCs were then plated onto a 24‐well plate at a density of 3 × 10^6^ ml^−1^ and cultured in RPMI 1640 medium supplemented with 10% FBS and 100 ng ml^−1^ M‐CSF (PeproTech, USA, cat. 300–25) for 7 days to obtain macrophages. Following this, the macrophages were polarized into M1 phenotype by stimulation with 100 ng ml^−1^ LPS and 20 ng ml^−1^ IFN‐γ.

### Transfection

The siRNA, miR‐31‐5p mimics, inhibitors, and their corresponding negative controls were designed and synthesized by Gene Pharma (Suzhou, China). PBMCs and THP‐1 derived macrophages were transfected with these RNAs at a final concentration of 300 and 100 nmol, respectively, using Lipofectamine 2000 Reagent (Thermo Fisher Scientific, Waltham, MA, USA) following the manufacturer's instructions. At 24 h post‐transfection, THP‐1 derived macrophages and PBMCs from SS dry eye patients were stimulated with LPS and IFN‐γ to induce M1 macrophage polarization, and PBMCs from model rabbits were collected and cocultured with irradiated pLGECs for subsequent experiments. All sequence information used in this study was shown in **Table** **1**.

**Table 1:** List of oligonucleotides used in this study.

Name	Sence Sequence (5^′^–3^′^)	Antisence Sequence (5^′^–3^′^)
Ctrl mimics	UUCUCCGAACGUGUCACGUTT	ACGUGACACGUUCGGAGAATT
Ctrl inhibitor	CAGUACUUUUGUGUAGUACAA	
Ctrl siRNA	UUCUCCGAACGUGUCACGUTT	ACGUGACACGUUCGGAGAATT
miR‐31‐5p‐mimics (rabbit)	AGGCAAGAUGCUGGCAUAGCUGU	AGCUAUGCCAGCAUCUUGCCUUU
FTO siRNA‐1 (rabbit)	GCUGAAAUAGGUGCUGCUUTT	AAGCAGCACCUAUUUCAGCTT
FTO‐siRNA‐2 (rabbit)	GCCAGUGUGCAUGGCAGAATT	UUCUGCCAUGCACACUGGCTT
miR‐31‐5p‐mimics (human)	AGGCAAGAUGCUGGCAUAGCU	CUAUGCCAGCAUCUUGCCUUU
P2RX7 siRNA (human)	CGAUGGACUUCACAGAUUU	AAAUCUGUGAAGUCCAUCG
miR‐31‐5p‐inhibitor (human)	AGCUAUGCCAGCAUCUUGCCU	
FTO siRNA (human)	AAAUAGCCGCUGCUUGUGAGA	UCUCACAAGCAGCGGCUAUUU

### Real‐Time qRT‐PCR and Western Blot

Total RNA from LGs or cells was extracted using EZ‐press RNA Purification Kit (EZBioscience, USA) according to the manufacturer's instructions. The first‐strand cDNA was synthesized with the reverse transcription kit (Thermo Fisher Scientific, USA) and the real‐time qRT‐PCR was performed using SYBR Green Master Mix (Thermo Fisher Scientific, USA) with a Roche LightCycler 480 II Analyzer. The relative gene expression was calculated using the 2 ^[ΔCt(control)–ΔCt(target)]^ method. Gene‐specific primers are listed in **Tables** **2**, **3**, **4**.

**Table 2:** Sequences of primers in this study for real‐time qRT‐PCR (rabbit).

Gene	Forward Primer Sequence (5^′^–3^′^)	Reverse Primer Sequence (5^′^–3^′^)
GAPDH	GGGTGGTGGACCTCATGGT	CGGTGGTTTGAGGGCTCTTA
Arg1	GAAGTAACTCGAACGGTGAACACA	TCCCGAGCAACTCCAAAAGA
CD206	CTGATAGATGGAGGGTGAGGTACA	CCAGATAGACGCATGCTGACTTC
TGF‐β	CAAGGACCTGGGCTGGAA	AGGCAGAAGTTGGCGTGGTA
IL‐10	GGCTGAGGCTGCGACAAT	TGCCTTGCTCTTGTTTTCACA
NOS2	TCCACCAGGAGATGCTCAACT	TGGGTTTTCCACGCCTCTAC
IL‐1β	CTCCTGCCAACCCTACAACAA	TCCAGAGCCACAACGACTGA
TNF‐α	AGCTTCTCGGGCCCTGAGT	CCACTTGCGGGTTTGCTACT
P2RX7	GCAGAAAGGGATGGATGGAC	CCTCGTGGTGTAGTTGTGGC
METTL3	ATTGAGGTAAAGCGAGGTCTCC	GCTTGGAATGGTCAGCATAGGT
METTL14	AACAATCCTGGCAAGACAA	CATCTGTGCTACGCTTCA
WTAP	CAACAACAGCAGGAGTCT	AGTCGCATTACAAGGATGT
FTO	TATCTCGCATCCTCATTGG	TTGACAAGCAGCACCTATT
pri‐miR‐31	TTAACTTGGATCTGGAGAGG	AACACATGGAGGAATGGTAT

**Table 3:** Sequences of primers in this study for real‐time qRT‐PCR (human).

Gene	Forward Primer Sequence (5^′^–3^′^)	Reverse Primer Sequence (5^′^–3^′^)
GAPDH	CTGGGCTACACTGAGCACC	AAGTGGTCGTTGAGGGCAATG
Arg1	GCGCCAAGTCCAGAACCA	CGTGGCTGTCCCTTTGAGAA
CD206	CGCTACTAGGCAATGCCAATG	GCAATCTGCGTACCACTTGTTT
IL‐10	TGAGAACAGCTGCACCCACTT	TCGGAGATCTCGAAGCATGTTA
NOS2	CCCCTTCAATGGCTGGTACA	GCGCTGGACGTCACAGAA
IL‐1β	TCAGCCAATCTTCATTGCTCAA	TGGCGAGCTCAGGTACTTCTG
TNF‐α	GCAGGTCTACTTTGGGATCATTG	GCGTTTGGGAAGGTTGGA
P2RX7	AGGGCGGAATAATGGGC	GGAAACTGTATTTGGGACGG
FTO	GTTCACAACCTCGGTTTAGTTC	CATCATCATTGTCCACATCGTC

**Table 4:** Gene‐specific primers used for stem‐loop q‐PCR.

	primer sequence(5^′^‐3^′^)		
Name	Reverse transcription	Forward	Reverse
ocu‐miR‐31‐5p	GTCGTATCCAGTGCAGGGTCCGAGGTATTCGCACTGGATACGACACAGCT	CGAGGCAAGATGCTGGCAT	AGTGCAGGGTCCGAGGTATT
hsa‐miR‐31‐5p	GTCGTATCCAGTGCAGGGTCCGAGGTATTCGCACTGGATACGACAGCTAT	GCGAGGCAAGATGCTGGC	AGTGCAGGGTCCGAGGTATT
hsa‐miR‐381‐3p	GTCGTATCCAGTGCAGGGTCCGAGGTATTCGCACTGGATACGACACAGAG	CGCGTATACAAGGGCAAGCT	AGTGCAGGGTCCGAGGTATT
hsa‐miR‐656‐3p	GTCGTATCCAGTGCAGGGTCCGAGGTATTCGCACTGGATACGACAGAGGT	GCGCGCGAATATTATACAGTCA	AGTGCAGGGTCCGAGGTATT
hsa‐miR‐432‐5p	GTCGTATCCAGTGCAGGGTCCGAGGTATTCGCACTGGATACGACCCACCC	CGCGTCTTGGAGTAGGTCATT	AGTGCAGGGTCCGAGGTATT
hsa‐miR‐34a‐5p	GTCGTATCCAGTGCAGGGTCCGAGGTATTCGCACTGGATACGACACAACC	CGCGTGGCAGTGTCTTAGCT	AGTGCAGGGTCCGAGGTATT
hsa‐miR‐219‐5p	GTCGTATCCAGTGCAGGGTCCGAGGTATTCGCACTGGATACGACAGAATT	CGCGTGATTGTCCAAACGC	AGTGCAGGGTCCGAGGTATT
U6	TTCACGAATTTGCGTGTCATC	CGCTTCGGCAGCACATATAC	TTCACGAATTTGCGTGTCATC

For western blot, cells or frozen tissues were lysed with RIPA lysis buffer (Solarbio, Beijing, China) containing protease inhibitor PMSF (Solarbio) and phosphatase protease inhibitor cocktail (Cell Signaling Technology, Danvers, Massachusetts, USA), and the protein concentration was quantified using the Bicinchoninic Acid Assay Kit (Solarbio). Equal amounts of proteins were separated by SDS‐PAGE and transferred onto polyvinylidene difluoride (PVDF) membrane. After blocking with 5% non‐fat milk, membranes were incubated with antibodies specific for Arg1 (1:500, Abcam, UK, cat. ab239731), NOS2 (1:500, R&D Systems, MAB9502‐SP, for rabbit sample detection; 1:1000, Cell Signaling Technology, USA, cat. #20609, for human sample detection), phospho‐p38, phospho‐JNK, phospho‐ERK (1:2000, Cell Signaling Technology, USA, cat. #9215, #9106, #9255), P2RX7 (1:1000, Cell Signaling Technology, USA, cat. #13809), DGCR8 (1:1000, Abcam, UK, cat. ab191875), FTO (1:1000, Cell Signaling Technology, USA, cat. # 31687) or β‐actin (1:2000, ZSGB‐BIO, China, cat. TA‐09) and detected using Tanon 4800 Multispectral Imaging System (Tanon Science & Technology, China).

### Luciferase Reporter Assay

First, the P2RX7 3^′^UTR‐ WT fragment or P2RX7 3^′^UTR‐ Mut fragment was amplified and cloned into pMIR‐Report vector (Promega, Madison, USA) as we described previously.^[^ ^55^, ^56^ ^]^ The sequences of primers used were as follows: P2RX7 3^′^UTR‐WT forward, 5^′^‐GGACTAGTCCCGTTTGGAGTCAGGATTTG‐3^′^; P2RX7 3^′^UTR‐WT reverse, 5^′^‐ CCCAAGCTTCAAACAGTCAAGCTAAACAAGAAT‐3^′^; P2RX7 3^′^UTR‐Mut forward, 5^′^‐ GGACTAGTCTGTGTCTGTGTAGACCGTTTTCAACTACTGCCTAAAGT‐3^′^; P2RX7 3^′^UTR‐Mut reverse, 5^′^‐ CCCAAGCTTTCCTGGGCGGTTACCACGGTCAGAG‐3^′^. The dual‐luciferase reporter plasmids for both the WT and Mut forms of pri‐miR‐31 were purchased from Hanbio. Specifically, pri‐miR‐31 sequence was cloned into a Pmir‐GLO dual luciferase expression vector. In the Mut plasmid, the adenosine base in the m6A consensus sequences was replaced with thymine to abolish the m6A modification. For luciferase reporter assay, HEK293T cells were cultured in the 96‐well plates at a density of 2.5 × 10^4^ cells/well. 24 h later, HEK293T cells were co‐transfected with indicated plasmids and oligonucleotides using Lipofectamine 2000 reagent (Thermo Fisher Scientific). 48 h later, the samples were collected, and the relative luciferase activity was detected by the Dual‐Luciferase Reporter Assay System (Promega) following the manufacturer's instruction.

### Immunofluorescence Staining

miR‐31‐5p mimics or control mimics transfected macrophages were fixed with 4% paraformaldehyde (Solarbio) for 15 min at room temperature. After blocking with 5% goat serum (Solarbio) for 1 h, cells were incubated with mouse anti‐Arg1 primary antibody (1:100, Abcam, UK, cat. ab239731) or mouse anti‐NOS2 primary antibody (1:250, R&D Systems, MAB9502‐SP) overnight at 4 °C. The next day, the cells were washed and incubated with goat anti‐mouse Alexa fluor647 (IgG H&L) secondary antibodies (1:500, Abcam, UK) and DAPI (0.5ug ml^−1^, Sigma–Aldrich, Merck) at room temperature for 1h. Fluorescence images were captured using a confocal fluorescence microscope (LSM800, Zeiss, Oberkochen, Germany).

### Bioinformatic Analysis

The miRanda database was used to identify the potential targets of miR‐31‐5p. The m6A sites of pri‐miR‐31 were analyzed using SRAMP (http://www.cuilab. cn/sramp). The DAVID database (https://david.ncifcrf.gov) was employed for KEGG pathway enrichment analysis. The target genes of miR‐31‐5p were mapped for the GO category: immune system process using Cytoscape software v3.7.0.

### MeRIP‐qPCR

Total RNA was extracted from FTO‐knockdown PBMCs and negative controls using the Universal RNA Purification Kit (EZBioscience, USA), and the Magna MeRIP m6A Kit (Millipore, Billerica, MA, USA; cat, 17–10499) was employed to determine the enrichment of m6A modifications on the pri‐miR‐31 transcript according to the manufacturer's instructions. Briefly, the anti‐m6A antibody was conjugated to protein A/G magnetic beads and mixed with 80 µg of total RNA in IP buffer. Then, the m6A‐modified RNA was eluted twice with 20 mm N6‐methyladenosine 5ʹ‐monophosphate sodium salt at 4 °C for 1h. Finally, real‐time qRT‐PCR was performed to quantify the abundance of m6A enrichment on pri‐miR‐31.

### RNA Stability Assay

PBMCs isolated from model rabbits were transfected with FTO siRNA or its negative controls for 24h. Following this, the cells were exposed to Actinomycin D (MedChemExpress, Monmouth Junction, NJ, USA) for 0, 3, 6, and 9 h at a final concentration of 5 µg ml^−1^. Subsequently, total RNA was extracted, and the relative level of pri‐miR‐31 in each group at the indicated time was analyzed by real‐time qRT‐PCR. The degradation rate (*k*) and half‐life (*t_1/2_*) of pri‐miR‐31 were estimated by the following equation:

(1) $$\ln\left(\frac{A_{t}}{A_{0}}\right)=-kt$$

(2) $$t_{1/2}=\frac{ln2}{k}$$

where *At* and *A0* represent the concentration of pri‐miR‐31 at time *t* and time *0*, and *t* is the transcription inhibition time.

### Co‐IP and RIP Analysis

Co‐IP experiments were performed in HEK293T and THP‐1 cell lines using Pierce Classic Magnetic IP/Co‐IP Kit (cat. 88 804; Thermo Fisher Scientific) according to the manufacturer's protocol. Briefly, whole cell lysates were incubated overnight at 4 °C with gentle shaking, using either 8 µg of anti‐DGCR8 antibody (Abcam, UK; cat. ab191875) or anti‐IgG antibody (Proteintech Group, Chicago, IL; cat. 30000‐0‐AP). Subsequently, the complex was incubated with protein A/G magnetic beads for 1 h at room temperature. After washing, the immune complex bound to the beads was dissociated using a low‐pH buffer for subsequent western blot analysis.

The Magna RIP kit (Millipore, Billerica, MA, USA; cat, 17–700) was utilized to conduct the RIP experiment. In briefly, HEK293T cells transfected with either FTO siRNA or negative controls were collected and lysed in RIP lysis buffer that contained both protease and RNase inhibitors. Then the lysate was incubated with magnetic beads coupled with the anti‐DGCR8 antibody at 4 °C overnight. Subsequently, the coprecipitated RNAs were isolated and subjected to real‐time qRT‐PCR analysis.

### Statistical Analysis

Data from at least three independent experiments were presented as mean ± SD and analyzed using GraphPad Prism 9.0 software. The normality of the data was evaluated by the Shapiro–Wilk test. Statistical significance between groups was determined using the Unpaired Student's *t*‐test, Mann–Whitney test, or one‐way/two‐way ANOVA, as appropriate. The indicated *p* values <0.05 were considered statistically significant. The correlation analysis was calculated by the Pearson correlation test. A correlation coefficient (*r* value) > 0 indicates a positive correlation between the two variables, whereas an *r* value < 0 signifies a negative correlation.

### Ethics Approval Statement

The human participants in this study were reviewed and approved by the Ethics Committee of Tianjin Medical University Eye Hospital. Written informed consent was obtained from all subjects. The animal study was reviewed and approved by the Animal Care and Use Committee of the Tianjin Medical University Eye Hospital.

## Conflict of Interest

The authors declare no conflict of interest.

## Author Contributions

L.Z. and X.L. contributed equally to this work. L.Z., X.J.L., and M.G. performed the experiments, X.L., J.C.Z., and R.X.L. assisted with data analysis and provided technical assistance. L.L., B.Y.M., and B.D. collected samples from clinical patients and analyzed the data. L.Z. wrote the manuscript. HN and RHW designed the research, supervised the overall project, wrote the manuscript, and provided financial support. All authors contributed to the article and approved the submitted version.

## Supporting information



Supporting Information

## Data Availability

The raw data supporting the conclusions of this article will be made available by the authors, without undue reservation.
