# Supplementary material for: M6A Modified miR‐31‐5p Suppresses M1 Macrophage Polarization and Autoimmune Dry Eye by Targeting P2RX7
Source: Adv Sci (Weinh). 2025 Mar 11;12(17):2415341. doi: 10.1002/advs.202415341 (PMC12061282; doi:10.1002/advs.202415341)
Supplement: Supplementary file 1 — Supporting Information [file ADVS-12-2415341-s001.docx]

Supporting Information

Title：M6A Modified miR-31-5p Suppresses M1 Macrophage Polarization and Autoimmune Dry eye by Targeting P2RX7

*Lu Zhao, Xuejia Li, Min Gao, Lin Liu, Binyun Ma, Xun Liu, Jiachen Zhang, Ruoxuan Liu, Bei Du, Ruihua Wei*, and Hong Nian**

Figure S1


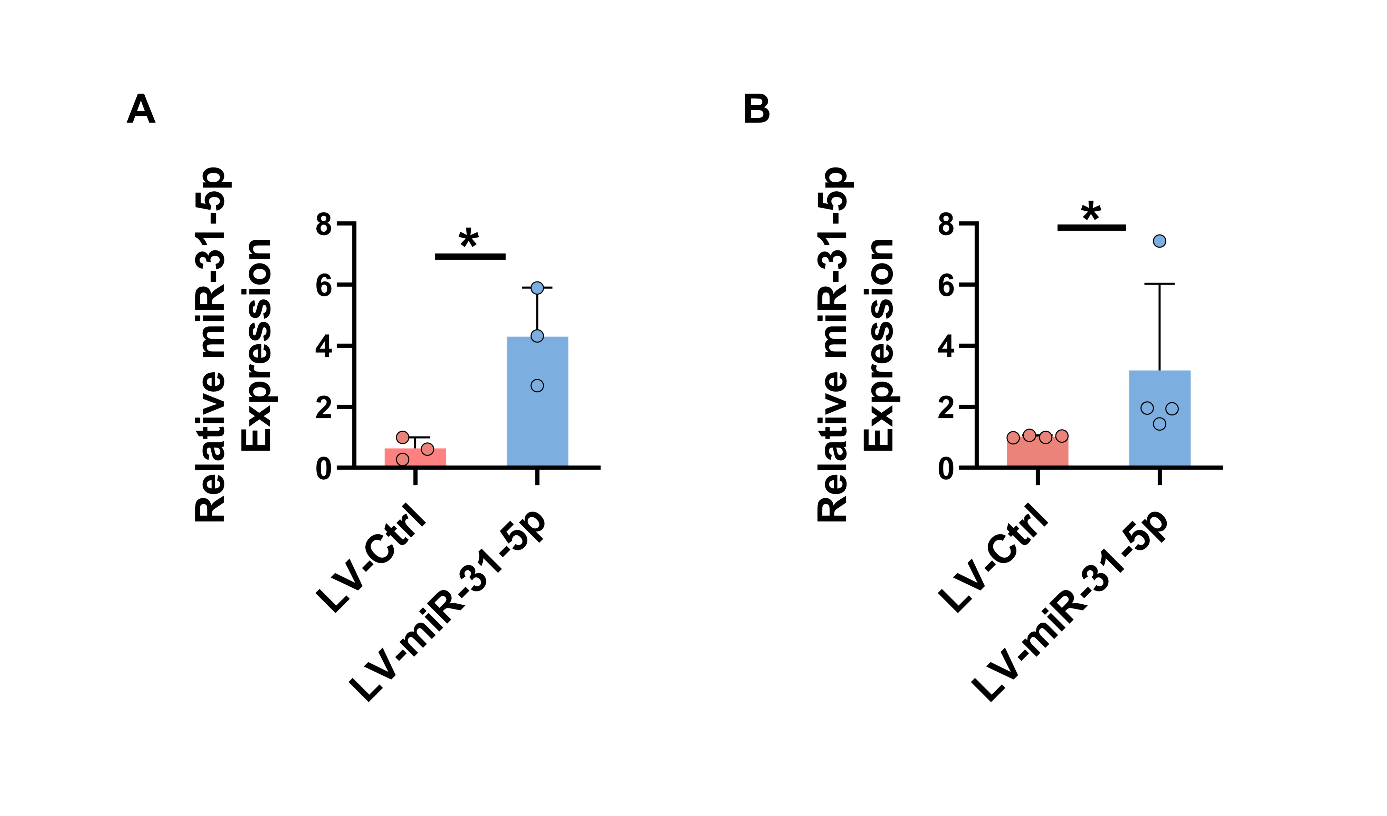


**Figure S1. MiR-31-5p is significantly upregulated in rabbits injected with miR-31-5p-overexpressing lentivirus.** (A) Rabbits were subconjunctivally injected with a single dose of miR-31-5p-overexpressing lentivirus at the time of disease induction (day 1 post transfer). The relative miR-31-5p level is shown. (B) A single dose of miR-31-5p-overexpressing lentivirus was injected subconjunctivally into rabbits after disease onset (day 15 after transfer). Real-time qRT-PCR analysis of miR-31-5p expression. Data were representative of at least three independent experiments and were analyzed by Unpaired Student’s t-test or Mann-Whitney U test. *P < 0.05.

Figure S2


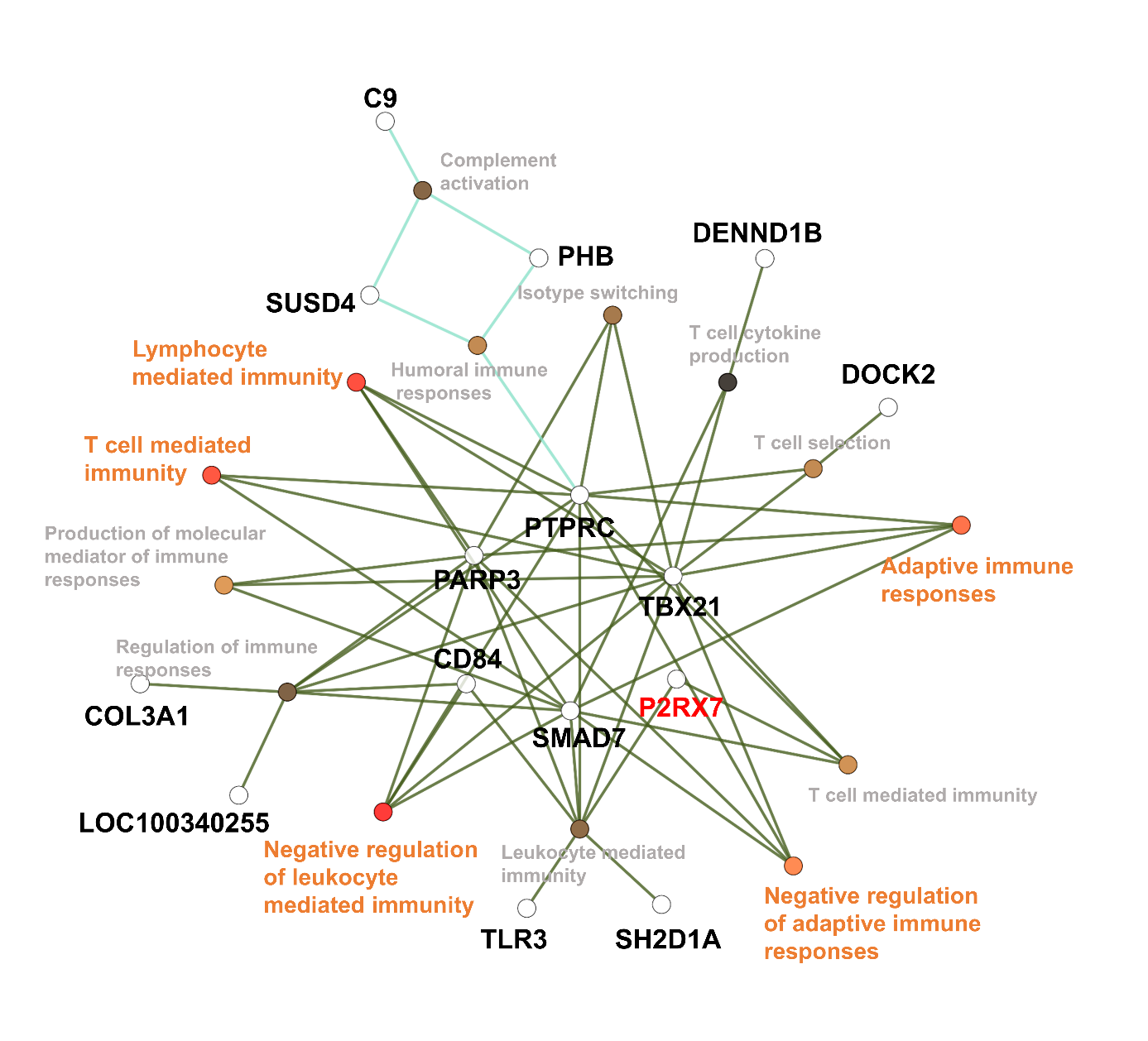


**Figure S2.** The target genes of miR-31-5p were mapped for the GO category: immune system process using Cytoscape software.
